# Macrophage-mediated tissue response evoked by subchronic inhalation of lead oxide nanoparticles is associated with the alteration of phospholipases C and cholesterol transporters

**DOI:** 10.1186/s12989-022-00494-7

**Published:** 2022-08-03

**Authors:** Tereza Smutná, Jana Dumková, Daniela Kristeková, Markéta Laštovičková, Adriena Jedličková, Lucie Vrlíková, Bohumil Dočekal, Lukáš Alexa, Hana Kotasová, Vendula Pelková, Zbyněk Večeřa, Kamil Křůmal, Jiří Petráš, Pavel Coufalík, Dalibor Všianský, Samuel Záchej, Dominik Pinkas, Jan Vondráček, Aleš Hampl, Pavel Mikuška, Marcela Buchtová

**Affiliations:** 1 Laboratory of Molecular Morphogenesis, Institute of Animal Physiology and Genetics, v.v.i., Czech Academy of Sciences, Veveří 97, 602 00 Brno, Czech Republic; 2 Department of Histology and Embryology, Faculty of Medicine, Masaryk University, 625 00 Brno, Czech Republic; 3 Department of Experimental Biology, Faculty of Science, Masaryk University, 625 00 Brno, Czech Republic; 4 Department of Environmental Analytical Chemistry, Institute of Analytical Chemistry, v.v.i., Czech Academy of Sciences, 602 00 Brno, Czech Republic; 5 Department of Cytokinetics, Institute of Biophysics, v.v.i., Czech Academy of Sciences, 612 65 Brno, Czech Republic; 6 Department of Geological Sciences, Faculty of Science, Masaryk University, 625 00 Brno, Czech Republic; 7 TESCAN Brno, s. r. o., 623 00 Brno, Czech Republic; 8 Electron Microscopy Core Facility of the Microscopy Centre, Institute of Molecular Genetics, v.v.i., Czech Academy of Sciences, 142 20, Prague, Czech Republic

**Keywords:** Lead oxide nanoparticles, Inhalation, Lung macrophages, Liver macrophages, Cholesterol metabolism

## Abstract

**Background:**

Inhalation of lead oxide nanoparticles (PbO NPs), which are emitted to the environment by high-temperature technological processes, heavily impairs target organs. These nanoparticles pass through the lung barrier and are distributed via the blood into secondary target organs, where they cause numerous pathological alterations. Here, we studied in detail, macrophages as specialized cells involved in the innate and adaptive immune response in selected target organs to unravel their potential involvement in reaction to subchronic PbO NP inhalation. In this context, we also tackled possible alterations in lipid uptake in the lungs and liver, which is usually associated with foam macrophage formation.

**Results:**

The histopathological analysis of PbO NP exposed lung revealed serious chronic inflammation of lung tissues. The number of total and foam macrophages was significantly increased in lung, and they contained numerous cholesterol crystals. PbO NP inhalation induced changes in expression of phospholipases C (PLC) as enzymes linked to macrophage-mediated inflammation in lungs. In the liver, the subchronic inhalation of PbO NPs caused predominantly hyperemia, microsteatosis or remodeling of the liver parenchyma, and the number of liver macrophages also significantly was increased. The gene and protein expression of a cholesterol transporter CD36, which is associated with lipid metabolism, was altered in the liver. The amount of selected cholesteryl esters (CE 16:0, CE 18:1, CE 20:4, CE 22:6) in liver tissue was decreased after subchronic PbO NP inhalation, while total and free cholesterol in liver tissue was slightly increased. Gene and protein expression of phospholipase PLCβ1 and receptor CD36 in human hepatocytes were affected also in in vitro experiments after acute PbO NP exposure. No microscopic or serious functional kidney alterations were detected after subchronic PbO NP exposure and CD68 positive cells were present in the physiological mode in its interstitial tissues.

**Conclusion:**

Our study revealed the association of increased cholesterol and lipid storage in targeted tissues with the alteration of scavenger receptors and phospholipases C after subchronic inhalation of PbO NPs and yet uncovered processes, which can contribute to steatosis in liver after metal nanoparticles exposure.

**Graphical abstract:**

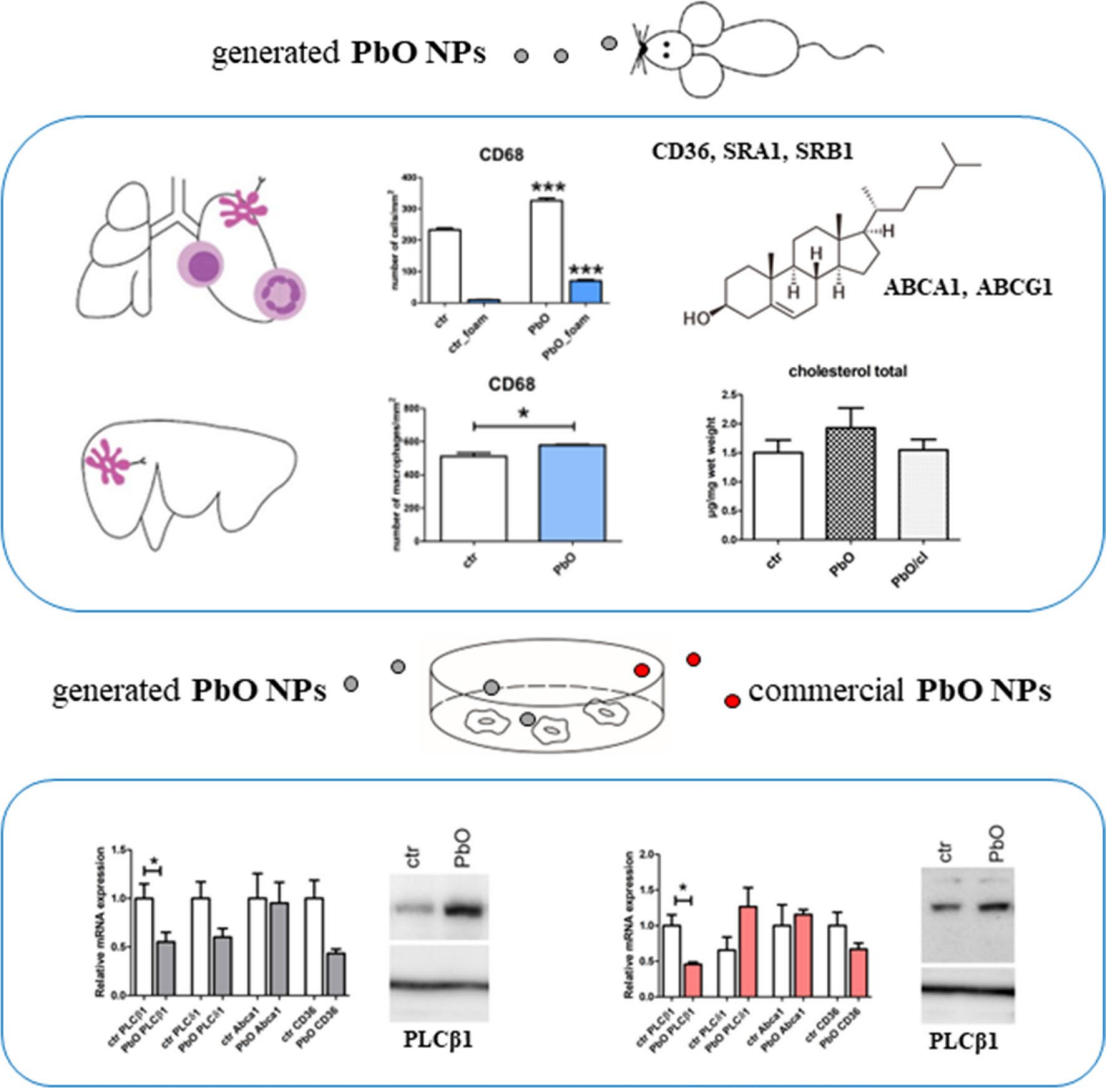

**Supplementary Information:**

The online version contains supplementary material available at 10.1186/s12989-022-00494-7.

## Background

Since the beginning of life, all living organisms are exposed to nanoparticles (NPs), objects which have at least one dimension ranging from 1 to 100 nm, produced by a variety of combustion processes, such as forest fires or volcanic activity. However, at the beginning of the industrial revolution, the volumes of nanoparticles released to environment increased dramatically, and this is currently being further aggravated with the expansion of nanotechnologies [49]. Nanoparticles can thus be divided into nanoparticles that arise as unwanted products from industrial processes (such as combustion processes) and nanoparticles prepared for defined applications. Indeed, nanoparticles produced in large amounts as negative outcomes contribute significantly to environmental pollution [49].

Lead toxicity has been known for many years, but lead is still widely used, for example, in batteries, various alloys, or ammunition. Lead is also used as a construction material in the chemical industry, an additive in engine lubricating oils, or part of X-ray protective suits [66]. The main sources of lead in the air are combustion processes, transport and combustion of fossil fuels and waste. Lead is also part of cigarette smoke. In the workplace, people come into contact with lead at most when they are melting or recycling batteries. Additionally, lead nanoparticles (Pb NPs) and especially their oxides (PbO NPs) are used mainly in the electrochemical industry, for example, as semiconductors in LEDs or infrared detectors [43, 86]. In the air, the main sources of lead particles are automobiles and air transport, lead smelters, and the rapidly evolving electrical industry [49]. The results of health risk assessment of urban aerosol confirmed important contribution of toxic elements such as Pb, Cr, As and Cd to the carcinogenic and non-carcinogenic health risk of both PM1 and PM2.5 aerosol [18].

The main routes of NP entry into the body are inhalation, transdermal entry, and ingestion [14]. Absorption of NPs through the skin is rather minimal as skin represents an effective barrier compared to much more permeable gastrointestinal tract and lungs [39]. The lungs are the most effective primary gateway for NPs [13]; therefore, we used this pathway for NPs exposure and mice were placed into nanoparticles in whole-body inhalation chambers as this unique system corresponds with real-life exposure scenario, where animals are exposed to NPs dispersed in the air. Airborne NPs can come into contact with various parts of the body. Additionally, the animals take care of their fur so that oral exposure to inhaled NPs can be even higher than in humans.

Previously, we focused on the effect of inhalation of PbO NPs as they can pose a serious threat to human health because of their size and specific characteristics [8, 43]. The negative effect of subchronic PbO NP inhalation on animals was confirmed by morphological and functional changes being observed predominantly in the lungs and liver [8, 24, 25]. As we observed an inflammatory response after subchronic PbO NP inhalation in lung tissue in our previous studies [24, 25], we aimed to continue our evaluation of the role of macrophages in this response. Macrophages are cells participating in all phases of the immune and inflammatory response, and they can be found in organs throughout the body. They represent specific immune cells responsible for identifying and removing foreign substances from the body and can be activated towards pro-inflammatory (M1) or anti-inflammatory (M2) phenotypes depending on the stimuli [21]. Members of phospholipases C (PLC) are involved in intracellular and intercellular signaling to macrophage-mediated inflammation [87]. There is a total of six classes of PLC enzymes, with PLC β, δ, and γ being expressed in M1 and M2 macrophages. Individual types of macrophages then express a specific combination of individual PLC. We have selected three isoforms of PLC: PLCβ1, PLCγ2, and PLCδ1 to study the effect of PbO NP inhalation on macrophage populations in the lung, liver, and kidney. PLCβ1 is expressed in both M1 and M2 macrophages, similar to PLCγ2, while PLCδ1 is expressed only in M1 macrophages [87].

Macrophages, which become overloaded with lipids (predominantly cholesterol) are called lipid-laden macrophages or foam cells. Foam macrophages are typically formed during pathological changes of vessels (atherosclerosis or arthritis), or after infections caused by pathogens, such as *Mycobacterium tuberculosis*, *Toxoplasma*, or *Chlamydia* [67, 71]. Further, the presence of foam macrophages in the lung is pathological phenomenon seen after prolonged exposure to metal NPs [2]. As lipid homeostasis plays a crucial role during the transformation of macrophages into foam cells, we have further focused on possible changes in lipid metabolism. Here, we focus on membrane-bound scavenger receptors (SR) binding many lipoproteins, which can be classified into several classes according to their sequences (classes A–J) [84]. Macrophages express these surface membrane receptors such as scavenger receptor A1 (SR-A1), which is responsible for cholesterol uptake or SR-B1 predominantly contributing to cholesterol efflux (transfer of cholesterol from cells to HDL) [15]. Other studies proposed SR-B1 as a receptor mediating both efflux and influx of cholesterol from and into cells [73] [84]. CD36 (SR-B2) is a macrophage receptor playing a role not only in macrophage uptake of oxLDL but also platelet activation and aggregation, apoptosis, or in inflammation as this receptor binds oxLDL, apoptotic cells and bacterial pathogens [84]. Interestingly, CD68 (SR-D1), used often as a marker of macrophages, is a transmembrane receptor also identified as an oxLDL binding protein [55]. Although CD68 is commonly discussed in immune responses; its role in oxLDL processing is poorly understood. As characteristic receptors responsible for cholesterol efflux from the cells we selected ATP-binding cassette (ABC) transporters represented by ABCA1, and ABCG1 [15].

The liver is the centrum of lipid metabolism; therefore, disorders of lipid storage or metabolism are often considered to be indicative of hepatotoxicity [10]. Previously, decreased level of total lipid and cholesterol concentrations was found in the blood upon 12-week subchronic exposure of rats to lead nanoparticles (10 nm and 30 nm PbS) together with morphological changes in the liver [1]. The prolonged abnormal retention of lipids in the liver resulted in macrovesicular or microvesicular steatosis leading to metabolic dysfunction, inflammation, and hepatic fibrosis [60]. In humans, exposure to other metals, such as cadmium, can cause modifications in lipid metabolism, including cholesterol; however, the underlying mechanisms are still not very clear [85].

Cholesterol exists in two basic forms—as free cholesterol and cholesteryl esters (CEs). Cholesteryl esters are formed by esterification of cholesterol with long-chain fatty acids. Free cholesterol is an important structural component of cell membranes, while cholesteryl esters are the main transport and storage form of cholesterol [29]. Therefore, both forms of cholesterol play an active part in metabolic pathways. In addition, the CE/free cholesterol ratio has been shown to be altered in some diseases, such as hepatocellular carcinoma [36] or cardiovascular disease [27]. Moreover, the molecular composition of CE species and changes in specific CE species may be related to the development of other diseases such as cystic fibrosis, Huntington disease, neurological disorders like multiple sclerosis or Alzheimer’s disease [12, 46, 68].

Therefore, in this study, we aim to follow the mechanisms of action of PbO NPs on cells, tissues, and organs in details with focus on processes potentially leading to the alteration of lipid metabolism.

## Results

**Fig. 1:**
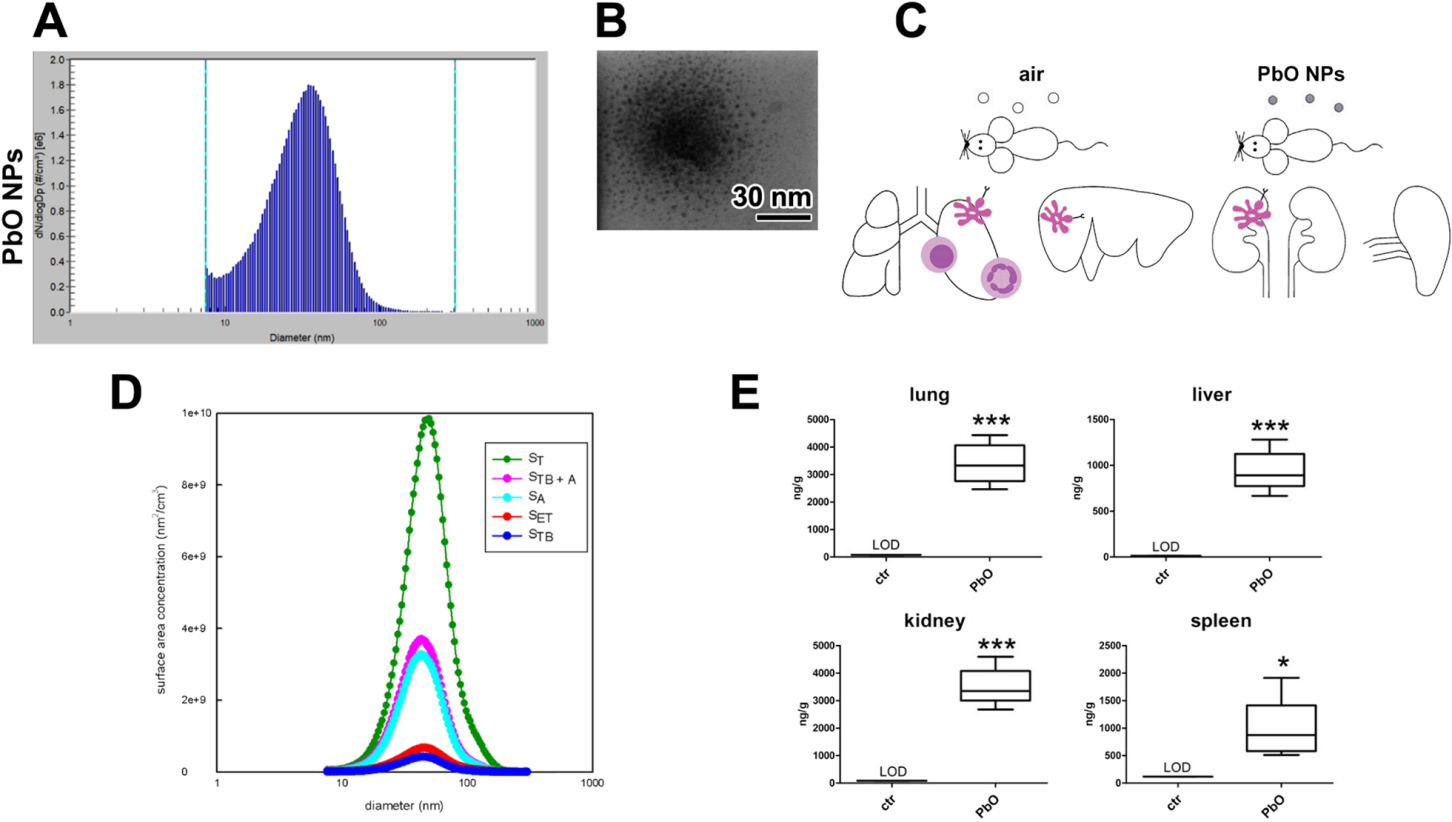
Characterization of PbO NPs. **A** Particle number-size distribution of PbO NPs in the inhalation chambers measured by Scanning Mobility Particle Sizer (SMPS). **B** STEM image of PbO NPs. **C** Design of the inhalation experiment. Symbols of light circle indicate clean air, and symbols of dark circles indicate PbO NPs. **D** Surface area of PbO NP size distribution (dS/dlogD_p_) calculated according to the ICRP deposition model. The surface area of fractions of PbO NPs deposited in the extrathoracic (S_ET_), tracheobronchiolar (S_TB_) and alveolar region (S_A_) of lungs, S_T_—the total surface area of generated PbO NPs, S_TB+A_—the lung-deposited surface area. **E** Analysis of Pb concentration (ng/g) in organs following 11 weeks of PbO NP inhalation. Limit of detection in the lung, liver, kidney, and spleen was 75, 13, 84, and 117 ng/g Pb, respectively. The graphs values denote average ± SD; *p < 0.05; ***p < 0.001 by unpaired t-test

The mice were exposed to PbO NPs in whole-body inhalation chambers where the concentration of NPs was 0.956 × 10^6^ NPs/cm^3^ (Fig. 1A–C). The estimated deposited dose over the 11-week inhalation period was 1.684 µg of PbO per gram of mouse body weight (Table 1). This value was calculated (Additional file 1) based on previously published methodology [5, 52, 54].

**Table 1:** Characterization of generated PbO NPs (1st experiment)

Characterization of PbO NPs	PbO
Number concentration	0.956 × 10^6^ NPs/cm^3^
Surface area	4.21 × 10^9^ nm^2^/cm^3^
Mode	34.6 nm
Geometric mean diameter	29.7 nm
Geometric standard deviation	1.69
Mass concentration	149.3 µg PbO/m^3^
Estimated deposited dose (after 11w)	1.684 µg PbO/g

The simulation of inhaled PbO NPs deposited in different parts of the respiratory tract of both human (Fig. 1D) and mouse was performed. For human, the deposition fractions for the extrathoracic, tracheobronchial, and alveolar region were calculated by the International Commission on Radiological Protection (ICRP) deposition model [33] while the deposition of PbO NPs in the mouse respiratory tract was calculated using a Multiple-Path Particle Dosimetry (MPPD) model [20, 56]. The total surface area of generated PbO NPs (i.e., 4.21 × 10^9^ nm^2^/cm^3^; S_T_) was calculated by Scanning Mobility Particle Sizer (SMPS) spectrometer software from a measured particle size distribution (Fig. 1A). The surface area of PbO NPs deposited in the human lung alveolar region (S_A_; 34.1% of the total surface area of PbO NPs) was found to be much higher than the surface area of NPs deposited in the extrathoracic (S_ET_; 7.34%) and tracheobronchial (S_TB_; 4.46%) regions. This finding was in contrast to the deposition in mouse respiratory tract, where the deposition in lung alveolar region (S_A_; 23.2% of the total surface area of PbO NPs) is comparable with the fraction deposited in the extrathoracic (S_ET_; 20.4%) region, while the fraction deposited in the tracheobronchial (S_TB_; 9.76%) region was lower. The sum of the surface area of the NPs deposited in the alveolar and tracheobronchial regions of the lungs forms so-called lung-deposited surface area (LDSA). The LDSA (S_TB__ + A_) corresponds to 38.5% and 33.0% of the total surface area of inhaled PbO NPs for human and mouse, respectively, while the sum of the surface area of the NPs deposited in the extrathoracic, tracheobronchial, and alveolar region forms 45.8% and 53.4% of the total surface area of inhaled PbO NPs for human and mouse, respectively.

The concentration of Pb was significantly higher after 11 weeks of PbO NP inhalation compared to the controls (Fig. 1E; Table 2) in all target organs studied here. The highest concentration of Pb was found in the kidney (3.5 µg/g tissue) and a comparable value of lead was detected in the lungs (3.4 µg/g tissue). The liver and spleen contained similar mean concentration of lead (0.9 µg/g tissue) that was about 3.5-fold lower than the Pb level in the kidney and lungs. Remarkably, the spleen displayed the highest differences in the concentration of Pb among the individual animals. In all control animals, the levels of Pb were always below the limit of detection (LOD) in all organs.

**Table 2:** Concentration of Pb (ng/g) in organs following 11 weeks of PbO NP inhalation

		ctr	PbO
Lung	Range	< LOD*	2463–4432
	Mean		**3393**
	SD		733
Liver	Range	< LOD*	667–1281
	Mean		**937**
	SD		222
Kidney	Range	< LOD*	2679–4592
	Mean		**3502**
	SD		693
Spleen	Range	< LOD*	512–1914
	Mean		**973**
	SD		551

Mean value is labelled by bold

Comparison of lead concentration in the lung, liver, kidney, and spleen in control and PbO NP inhaling animals. Limit of detection in the lung, liver, kidney, and spleen was 75, 13, 84, and 117 ng/g Pb, respectively

Next, we compared Pb mass (uptake) in individual analysed organs. This analysis uncovered the highest content of Pb again in kidneys (1.58 µg Pb; 2.8% in body), followed by similar amount of Pb in livers (1.56 µg Pb; 2.8% in body), lungs contained about 1.04 µg of Pb (1.9%), and the spleen 0.11 µg Pb (0.2%).

The total bodyweight of mice was not changed after 11-week PbO NP inhalation; however, the lung weight coefficient significantly increased in PbO NP animals (*p* < 0.05, Fig. S1). The weight coefficients of organs were expressed as wet weight of the organ (g)/dead body weight (g) × 100. The weight coefficient of kidney was also increased in PbO NP animals (*p* < 0.05 in the case of right kidney, there was no significant difference for the left kidney). The other organ weight coefficients for liver and spleen were not significantly altered after PbO NP inhalation.

### Subchronic PbO NP exposure caused only minor changes to the kidney parenchyma, while blood vessels contained large lipid droplets

**Fig. 2:**
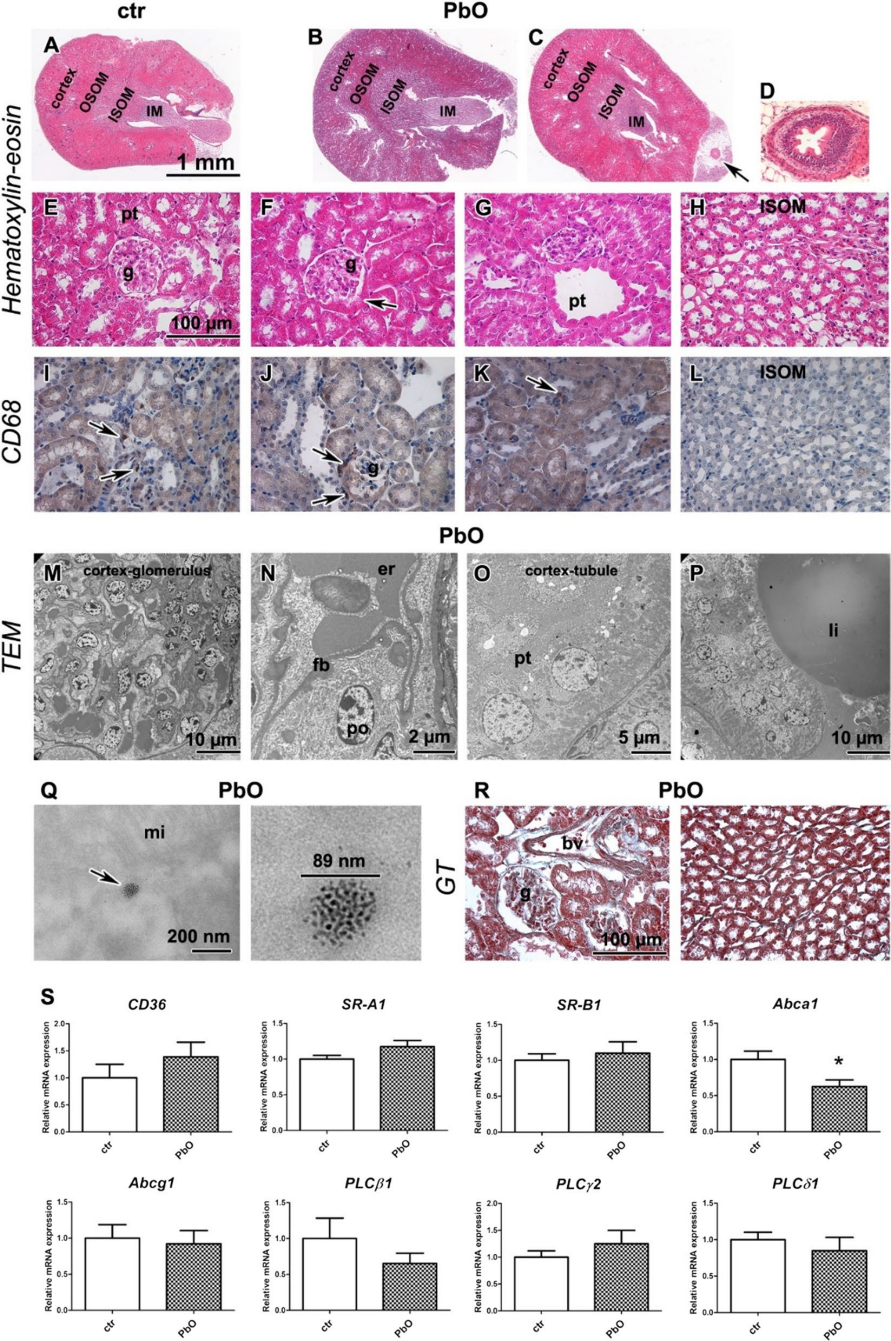
Kidney after 11-week PbO NP inhalation. **A**–**C** Kidney in overview image in HE staining (cortex, outer medulla divided into outer and inner stripe—OSOM, ISOM, inner medulla—IM) in control (**A**) and PbO NP exposed animals (**B**, **C**), arrow shows ureter. Scale bar in panels A-C = 1 mm. **D** Ureter in detail. **E** Kidney cortex of control animals with glomerulus (g) and proximal (pt) and distal tubules. **F**, **G** Kidney of PbO NPs treated samples exhibits metaplasia (*arrow*) of glomerular (g) parietal epithelium of Bowman’s capsule (**F**) or dilatation of proximal tubules (pt, G). **H** Kidney medulla without pathological alternations after exposure to PbO NPs. **I**–**L** Detection of CD68-positive cells (marker of macrophages) in kidney (arrows). Scale bar in panels = 100 µm. **M**–**Q** TEM images of kidney after inhalation of PbO NPs. **M** Renal glomerulus (glo) of characteristic apperance. N) typical podocyte (po) with pedicles, capillary with erythrocytes (er) and filtration barrier (fb) of kidney without pathological alteration. **O** Proximal tubule (pt) without damage. **P** Large lipid vacuole (li) present in blood vessel. **Q** Agglomerate of PbO nanoparticles in the epithelial cell of PT (arrow) next to mitochondria (mi). Scale bars are displayed individually for each picture. **R** Collagen fibers stained with Green Trichrome (GT) were found predominantly around blood vessels (bv). Scale bar in panels = 100 µm. **S** Gene expression of receptors *CD36*, *SR-A1*, *Abca1*, *Abcg1*, *SR-B1*, and phospholipases C after PbO NP inhalation. The graphs values indicate average ± SD; *p < 0.05 by unpaired t-test

As kidneys contained the highest concentration of lead after the 11-week PbO NPs, their exposure was the greatest from all analysed organs. However, only minor morphological changes were observed in the kidney (Fig. 2; Table S1). The glomerular metaplasia (Fig. 2F) was unexpectedly observed in both controls and PbO NP exposed animals, the prominent dilatation of proximal tubules was observed in one animal exposed to PbO NPs (Fig. 2G), and mild inflammatory perivascular cell infiltrates were irregularly located in kidney cortex in another PbO NP exposed animal (data not shown in figure). The kidney medulla did not exhibit any pathological features (Fig. 2H, L). In both glomerular and tubular compartments of kidney, the CD68-positive macrophages were scarce, with no difference between the control and PbO NP inhaling animals (Fig. 2I–L). The amount of collagen fibres visualized by Green Trichrome in PbO NP inhaling animals was similar to the controls (Fig. 2R).

At the ultrastructural level, the glomerular and tubular parts of nephrons were without alterations (Fig. 2M–O). The kidney filtration barrier exhibited a characteristic physiological appearance (Fig. 2N). Interestingly, large lipid vacuoles were present in some kidney blood vessels (Fig. 2P). The renal tubules closely attached to such vessels were compressed and displayed damage to their epithelial lining. Accumulations of nanoparticles were seen in the epithelial cells of proximal tubules (Fig. 2Q).

Next, we analyzed the mRNA expression of membrane receptors *CD36*, *SR-A1*, *Abca1*, *Abcg1*, and *SR-B1* (Fig. 2S). In the kidney, only expression of *Abca1* mRNA was significantly decreased (*p* < 0.01, Fig. 2S), the expression of other receptors remained at about the same level as in the controls. The expression of phospholipases *PLCβ1*, *PLCγ2*, and *PLCδ1* mRNAs in the kidney did not exhibit any significant changes upon the exposure of animals to PbO NPs (Fig. 2S).

### Inhalation of PbO NPs caused chronic inflammation in the lungs accompanied by massive cell infiltrates with abundant foam macrophages

**Fig. 3:**
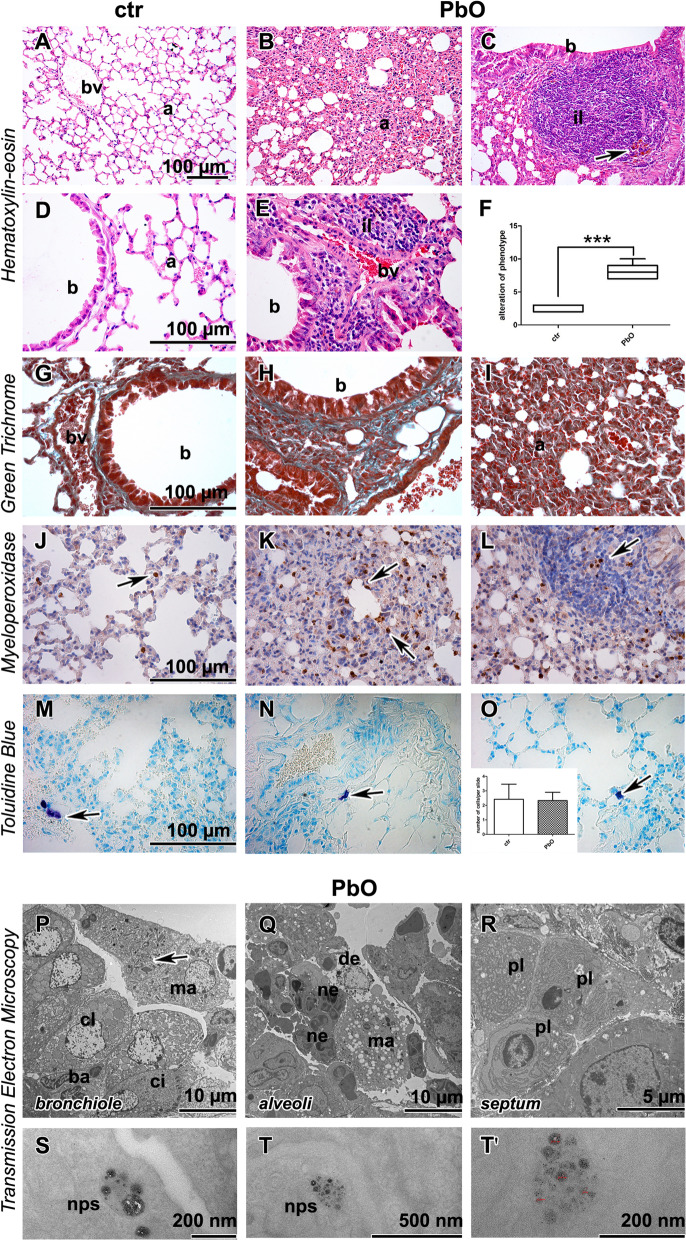
Lung after 11-week PbO NP inhalation. **A**, **D** Lungs in control animals without alternations. **B**, **C**, **E** Exposure to PbO NPs caused remodeling of lung tissue in alveolar areas (a). There are peribronchiolar (b) or perivascular (bv) inflammatory infiltrates of leukocytes (il) after PbO NPs inhalation. Arrow shows hemosiderin. **F** Evaluation of histopathological changes after 11 weeks of lead oxide nanoparticle inhalation according to the Table S1. The graphs values denote average ± SD; ***p < 0.001 by unpaired t-test. **G**–**I** Amount of collagen fibres (green) is not changed after inhalation of PbO NPs. Collagen fibers are around blood vessels (bv) and bronchioles (b). There are not any collagen fibers in alveolar areas despite serious remodeling. **J**–**L** MPO detection in lung tissue. Arrows display myeloperoxidase-positive cells—neutrophils. **M**–**O** Mastocytes (*arrows*) in lungs, insert displays the number of mastocytes per slide. Scale bar in all panels = 100 µm. **P**–**R** The ultrastructural morphology of the lung tissue with inflammatory features. **P** Terminal bronchiole lined with secretory club (cl), basal (ba) and ciliated (ci) cells; macrophage (ma) with cholesterol crystals inside (*arrow*) the lumen. **Q** Numerous macrophages (ma), neutrophils (ne) and abundant cell debris (de) in lung alveoli. **R** Clump of plasma cells (pl) around vessel in alveolar septum. **S**, **T**, **T′** Endosomes with PbO nanoparticles (nps) in pneumocyte type I. Scale bars are displayed individually for each picture

After 11-week PbO NP inhalation, histopathological analysis of the lung revealed numerous changes indicating severe tissue damage (Fig. 3A–F; Table S2). The lungs exhibited remodelling of tissue, bronchiolitis and alveolitis, atelectasis, hyperemia and dilated blood vessels, alveolar emphysema, thickened septa, increased number of cells in the interstitium, and occasionally haemorrhage (Fig. 3B, C, E, F). Siderophages were also found in lung parenchyma (Fig. 3C). Moreover, inflammatory infiltrates were seen around bronchioles and blood vessels. The histopathological changes to lungs were all statistically significant (*p* < 0.001) when compared to clean air-inhaling controls (Table S2). Severe morphological alterations of the lungs were also observed at ultrastructural level (Fig. 3P–R). Necrotic bronchiolar cells, bronchioles with accumulated neutrophils and macrophages, alveoli with cellular debris and inflammatory cells, and damaged membranes of lung cells, completed the image of deviations in lung tissues. Expectably, Green Trichrome staining confirmed collagen fibres in the walls of the vessels and bronchioles in both control and PbO NP exposed animals. However, collagen fibres as well as other signs of fibrosis were absent in alveolar parenchyma after PbO NP exposure (Fig. 3G–I).

As 11-week inhalation have produced large inflammatory infiltrates with abundant macrophages in lung tissue (Table S2), we then focused on observed inflammatory changes. Neutrophilic granulocytes, macrophages, mast cells, and lymphocytes were the main groups of the immune cells found in PbO NP exposed animals. Lymphocytes were dominant cells present in lung infiltrates (Fig. 3C, E). The number of neutrophils (visualized as MPO-positive cells) was increased in PbO NP exposed animals (Fig. 3J–L), and similarly to lymphocytes, they accumulated predominantly in lung infiltrates. Additionally, neutrophils were observed in the bronchioles and within alveolar spaces after PbO NP inhalation. The number of mastocytes (as visualised by Toluidine Blue staining) was not significantly altered after 11-week PbO NP inhalation (Fig. 3M–O). Clusters of plasma cells (mature B-cells producing immunoglobulins) were found in vicinity of blood vessels and bronchioles (Fig. 3R).

Besides unravelling subcellular damage, TEM also confirmed the presence of metal NPs in the lungs after PbO NP inhalation, with lead nanoparticles accumulating predominantly in the endosomes of alveolar epithelial cells I (Fig. 3S–T′) but not in alveolar epithelial type II cells (granular pneumocytes, PII). Nanoparticles formed clusters within cytoplasmic vesicles, which were well-separated from each other, and which exhibited different sizes corresponding to the range of diameters of the generated nanoparticles (Fig. 1A). The content of the vesicles, completely enveloped by cytoplasmic membrane, was more electron-dense than the surrounding cell cytoplasm. Furthermore, nanoparticles were also found in phagosomes of lung macrophages and neutrophils. Similar to membranous pneumocytes, nanoparticles in macrophages formed clusters within phagosomes, they were well-separated from each other, exhibited different sizes, and were often located adjacent to the vesicular membrane.

**Fig. 4:**
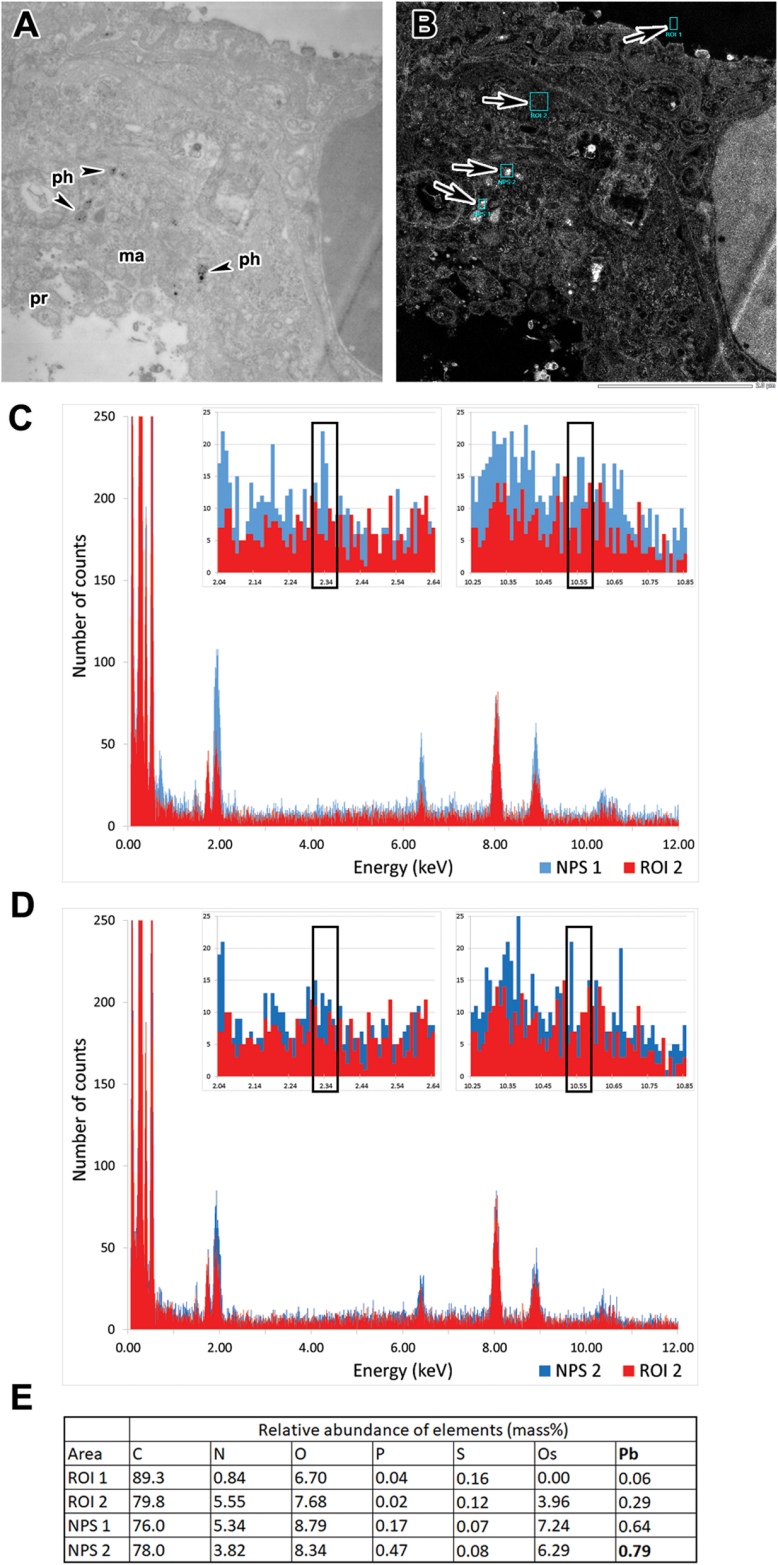
Detection of Pb content in lung nanoparticles by EDS. **A**, **B** STEM images of ultrathin sections collected with brightfield detector (**A**) with detecting angle 9.8 mrad and HAADF detector (**B**) detecting angle of 24.4–89.4 mrad. Alveolar macrophage (ma) with cytoplasmic processes (pr) and phagosomes (ph) with nanoparticles (*arrowheads*) in lung tissue after PbO NP inhalation. Cyan rectangles on (**B**) outline areas from which EDS spectra were recorded with ROI 1 being empty resin, ROI 2 being cytoplasm without any dense objects and NPS 1 and 2 marking phagosomes with nanoparticles. **C**, **D** EDS spectra with spectrum of cytoplasm (ROI 2) in red and spectra of NPS 1 and 2, respectively, in blue. Inserts show details of measured spectra with rectangles marking major peaks Pb Ma at 2.34 keV and Pb La at 10.55 keV. **E** Table summarizes estimates of relative mass of different elements in outlined regions of interest based on standard-less quantitative analysis

EDS measurements confirmed Pb content in the measured vesicles, marked “NPS 1” (Fig. 4A–C, outlined on Fig. 4B). The signal was weak and close to the detection limit; however, it was very specific, confirmed by detection of both Ma and La peaks at 2.34 and 10.55 keV respectively. While individual peaks were not apparent in spectra of NPS 2 (Fig. 4D), Zeta factor standardless quantitative analysis still uncovered elevated amounts of Pb in both vesicles compared to cytoplasm of the same cell—0.79 and 0.64 mass% for NPS 1 and NPS 2 respectively compared to 0.29 mass% in cytoplasm in ROI 2 or 0.06 mass% in empty resin (Fig. 4E).

### Increased numbers of foam macrophages were associated with altered expression of phospholipases C

**Fig. 5:**
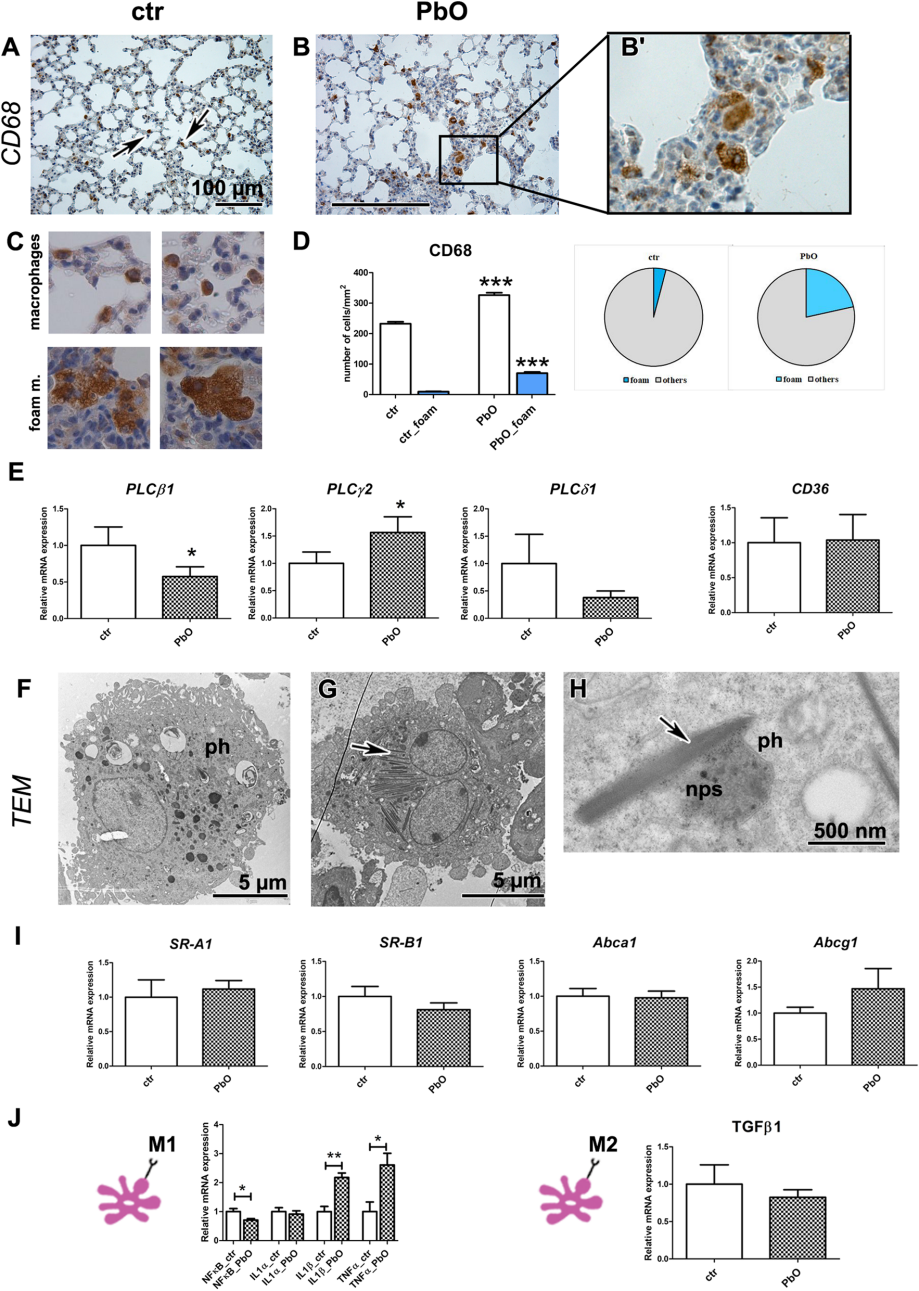
Lung macrophages after 11-week PbO NP inhalation. **A**, **B**, **B′** Detection of CD68-positive cells (marker of macrophages) in lungs (arrows). Scale bar in panels = 100 µm. **C** CD68-positive cells labelled as typical macrophages (two upper images) or foam macrophages (two lower images) at the same magnification in the slides. Differences in size and cytoplasm morphology of typical or foam macrophages are well distinguishable. **D** The number of macrophages, including foam macrophages, was significantly increased compared with the control group (the graphs values indicate average ± SD; ***p < 0.001 by unpaired t-test. **E** Gene expression of phospholipases *C*, and receptor *CD36* after PbO NP inhalation. The graphs values indicate average ± SD; *p < 0.05 compared with the corresponding control group (ctr) by unpaired t-test. **F**, **G** TEM images of foam alveolar macrophages with phagosomes (ph) in PbO NP group of animals. Arrow indicates cholesterol crystals in cytoplasm of macrophage. **H** Close interaction between phagosome (ph) with nanoparticles (nps) and cholesterol crystal (arrow). Scale bars are displayed individually for each picture. **I** Gene expression of receptors *SR-A1*, *SR-B1*, *Abca1* and *Abcg1* after PbO NP inhalation. The graphs values indicate average ± SD. **J** Gene expression of selected markers specific for M1 or M2 macrophage populations. The graphs values indicate average ± SD; *p < 0.05; **p < 0.01 compared with the corresponding control group (ctr) by unpaired t-test

Next, we focused on lung macrophages and their signalling since these cells are instrumental for immune and inflammatory response of this organ [70]. In the animals exposed to PbO NPs, both regular and foam macrophages were accumulated in the alveolar spaces, bronchioles, and interalveolar septa, with this accumulation being statistically significant (*p* < 0.001 for both regular and foam macrophages) as demonstrated by quantification of CD68-positive cells (marker of macrophages) (Fig. 5A–D; Table S3).

PLC signalling regulates a macrophage-mediated inflammatory response in lungs; therefore, we analysed its possible changes after exposure to PbO NPs. The expression of *PLCβ1* mRNA was significantly downregulated in the lungs after PbO NP inhalation (*p* < 0.05, Fig. 5E). Phospholipase PLCγ, which controls the maturation and function of B and T lymphocytes (PLCγ1 for T-cells and PLCγ2 for B-cells) [58], was affected after subchronic PbO NP inhalation, where the expression of *PLCγ2* mRNA was statistically significantly upregulated (*p* < 0.05, Fig. 5E). The effect of PLCγ2 upregulation on B-cells can explain the high accumulation of lymphocytes observed after PbO NP inhalation in lungs (Fig. 3C, E, R). On the other hand, phospholipase PLCδ1 affects the macrophage function by different mechanism compared to the above mentioned PLC members, as it negatively regulates inflammatory response in macrophages [38]. In consonance with this fact, *PLCδ1* mRNA was slightly (statistically non significantly) decreased in animals exposed to PbO NPs (Fig. 5E).

A large number of macrophages exhibited the appearance of foam cells with many cholesterol crystals and lipid droplets in their cytoplasm, as shown by TEM (Fig. 5F–H). Interestingly, the mRNA levels of cholesterol receptors *CD36*, *SR-A1*, *Abca1*, *Abcg1*, and *SR-B1* remained mostly unaffected in animals exposed to PbO NP (Fig. 5E, I). The only exception was statistically nonsignificant increase in mRNA of *Abcg1* (Fig. 5I).

Macrophages M1 (pro-inflammatory) release various cytokines and chemokines, incl. IL-1α, IL-1β, TNFα; while M2 macrophages (anti-inflammatory) produce e.g., TGF-β, IL-10, CCL1 [83]. Key transcription factor of M1 population *NF-κB* was downregulated (p < 0.05) after subchronic PbO NP inhalation. Further, PbO NP inhalation induced a statistically significant upregulation of expression of *IL-1β* mRNA (p < 0.01), and *TNFα* mRNA (p < 0.05) as markers of M1 macrophages (Fig. 5J). Expression of *IL-1α* mRNA was not significantly altered after PbO NP inhalation, similarly as *TGF-β* mRNA, the factor typical for M2 macrophages (Fig. 5J).

### Lead nanoparticles were detected in hepatocytes by energy dispersive X-ray (EDX) analysis

**Fig. 6:**
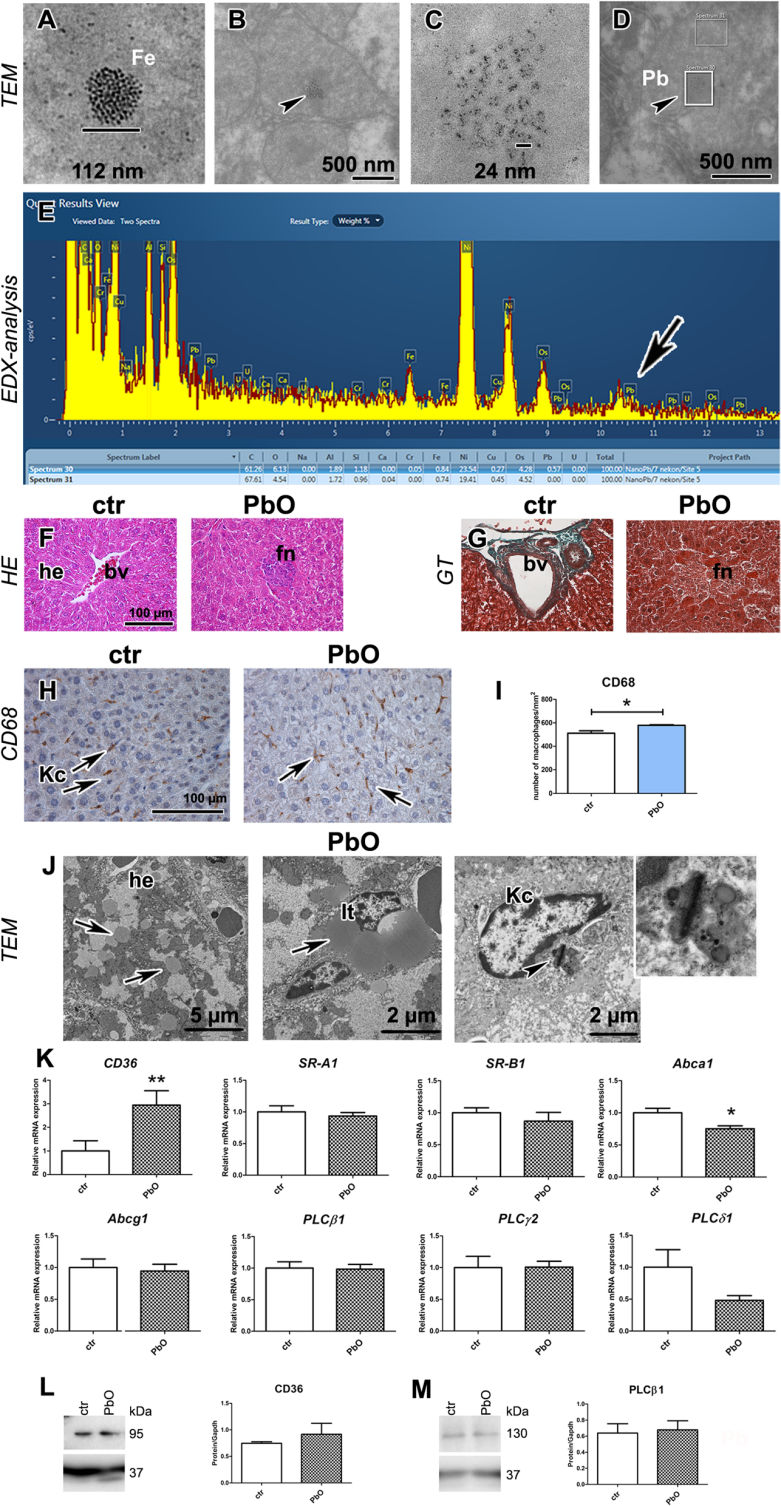
Liver after 11-week PbO NP inhalation. **A** Ferritin agglomerate (sized 112 nm). **B** Hepatocyte mitochondria (mi) with agglomerates of PbO nanoparticles (*arrowheads*) surrounded with electron-dense matrix. **C** Detail of agglomerates of PbO nanoparticles (size range 10–40 nm). **D** Hepatocyte mitochondrion with ROI windows analyzed inside. **E** SEM in transmission mode, using TESCAN RSTEM detector, and energy-dispersive *X*-ray spectroscopy (X-EDS) of lead treated samples of liver. Data obtained from two ROI windows analyzed inside hepatocyte mitochondria (**D**—with and without NPs). Spectra were compared with a reference sample and analyzed using Oxford AZtec. Presence of Pb was confirmed by comparing reference Spectrum 31 (without NPs) and non-reference Spectrum 30 (with NPs, *arrowhead* on image **D**). The peak displays increased signal at a spectral position of Pb (*arrow* on graph **E**). Nickel was observed during analysis as it was issued from the grid and osmium from post-fixation. **F** Liver in control and PbO NP treated animals (HE staining); *bv* blood vessels, *he* hepatocytes, *fn* focal necrosis. Scale bar in all panels = 100 µm. **G** Collagen fibers (green) in liver are around blood vessel (bv) in both groups (GT staining). There is no presence of fibrosis in focal necrosis (*fn*). Scale bar in all panels = 100 µm. **H** Detection of CD68-positive cells (marker of macrophages, *Kc*, Kupffer cells) in liver (arrows). Scale bar in panels = 100 µm. **I** The number of macrophages was significantly increased compared with the control group (the graphs values indicate average ± SD; *p < 0.05). **J** TEM images of hepatocytes (he) with lipid droplets (*arrows*), Ito cell (It) with lipid droplets (*arrow*), and Kupffer cell (Kc) with phagosomes. Arrowhead displays cholesterol crystal in phagosome. Scale bars are displayed individually for each picture. **K** Gene expression of receptors *CD36*, *SR-A1*, *SR-B1*, *Abca1*, *Abcg1* and phospholipases C after PbO NP inhalation. The graphs values indicate average ± SD; *p < 0.05, **p < 0.01 by unpaired t-test. **L** Protein expression level of receptor CD36 after PbO NP inhalation. The quantitative comparison of CD36 level was normalized to GAPDH level. **M** Protein expression level of phospholipase PLCβ1 after PbO NPs treatment. The quantitative comparison of PLCβ1 level was normalized to GAPDH level

Hepatocytes are major liver cells that physiologically store iron, one of the biogenic metal elements. Ultrastructurally, the particles of iron can be seen in the hepatocyte cytoplasm in the form of individual local agglomerates of small primary particles [32]. As lead nanoparticles can also form agglomerates (size range approximately 40–50 nm) of primary particles of 0.4–0.5 nm in diameter, we decided to perform EDX analysis to identify the chemical composition of particles observed by TEM and possibly distinguish between lead and iron particles (Fig. 6A–E).

Agglomerates of particles were randomly distributed in hepatocyte cytoplasm and mitochondria. Within selected mitochondrion with agglomerates of particles, we analyzed two different region of interest (ROI) windows (with and without agglomerates) by EDX. Spectra were compared with a reference sample and analyzed using Oxford AZtec. The presence of Pb was confirmed by comparing the reference Spectrum 31 (Fig. 6D, without NPs) and non-reference Spectrum 30 (Fig. 6D, with NPs) by increased signal at a spectral position of Pb. Therefore, hepatocyte mitochondria contained agglomerates of lead nanoparticles (Fig. 6E).

### Microvesicular steatosis and increased cholesterol uptake develop in the liver upon PbO NP inhalation

The liver represents the central organ of the main metabolic processes. We observed that 11 weeks long PbO NPs inhalation caused major hemostasia and sinusoidal damage, focal necrosis, and liver remodelling (Fig. 6F, G; Fig. S2, Table S4). Animals exposed to PbO NPs exhibited appearance of numerous macrophages in their liver parenchyma as well as necrotic foci with inflammatory leukocyte infiltrates, predominantly lymphocytes and neutrophils. Infiltration of immune cells was also observed in the portal areas. However, when histopathologically scored, the morphological alteration in liver parenchyma in animals exposed to PbO NPs were not statistically significant in comparison to controls (Table S4, Fig. S2). As visualized by Green Trichrome staining, the quantity of collagen fibres in the portal areas, walls of blood vessels, and bile ducts were similar in the livers of control animals and animals exposed to PbO NPs (Fig. 6G), documenting the absence of liver fibrosis.

The subchronic 11-weeks exposure to PbO NPs also caused microvesicular steatosis in their livers (Fig. 6J) with numerous lipid droplets being scattered in cytosol of hepatocytes, as shown by TEM. Ito cells displayed large lipid vacuoles (Fig. 6J). Next, we analysed receptors responsible for cholesterol uptake (CD36, and SR-A1) and cholesterol efflux (ABCA1, ABCG1, and SR-B1) in the liver. In contrast to lungs, mRNA expression of scavenger receptor *CD36* and transporter *Abca1* were significantly deregulated in the liver (*p* < 0.01 and *p* < 0.05, increased gene expression of *CD36* for cholesterol uptake, and reduced gene expression of *Abca1* for cholesterol efflux, respectively, Fig. 6K). The mRNA expression of other scavenger receptors remained unchanged. Protein level of receptor CD36 was also upregulated; however, this difference was not statistically significant after 11-week PbO NP inhalation, when compared to control samples, probably due to a higher variability in the Pb exposed group (Fig. 6L).

Taken together, PbO NP inhalation caused both morphological and functional alterations to liver, including the imbalance of lipid-associated molecules.

### Inhalation of PbO NPs caused alterations of liver macrophages number

Liver macrophages, called Kupffer cells, are resident cells localized along liver sinusoid endothelial cells. We used immunohistochemical detection of CD68 to visualize Kupffer cells in the liver (Fig. 6H; Table S5). The number of hepatic macrophages was significantly increased (*p* < 0.05) after 11-week inhalation of PbO NPs (Fig. 6I). The presence of cholesterol crystal was captured by TEM in cytoplasm of Kupffer cell (Fig. 6J). We also measured expression of PLC genes in the liver. Upon the exposure to PbO NPs, the levels of mRNAs for *PLCβ1* and *PLCγ2* remained unchanged, while the level of *PLCδ1* became modestly lowered (Fig. 6K). Similarly, the protein expression level of phospholipase PLCβ1 was not significantly altered by 11-week PbO NP inhalation (Fig. 6M).

### Alteration of cholesterol and cholesteryl esters in liver upon PbO NP inhalation

**Fig. 7:**
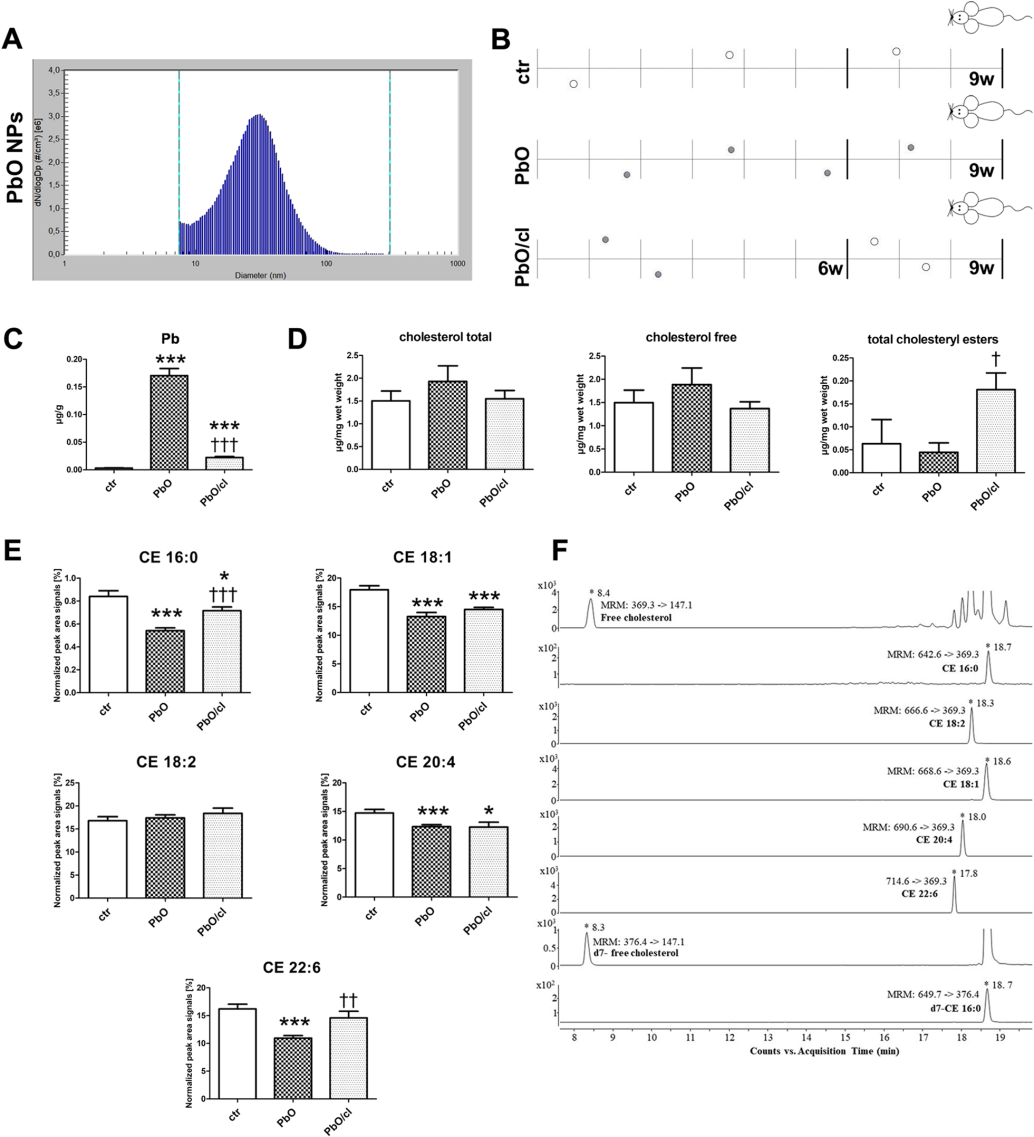
Cholesterol and cholesteryl esters in liver after 9-week PbO NP inhalation. **A** Particle number-size distribution of PbO NPs in the inhalation chambers measured by Scanning Mobility Particle Sizer (SMPS) in the 2nd experiment. **B** Design of the inhalation experiment. One group of animals inhaled clean air (ctr) for a period up to 9 weeks, the second group inhaled air with PbO NPs (PbO), and the third group inhaled air with PbO NPs for 6 weeks and thereafter clean air for following 3 weeks (PbO/cl—clearance group). Symbols of light circle indicate clean air, and symbols of dark circles indicate PbO NPs. **C** Analysis of Pb concentration (µg/g) in blood. Limit of detection in the blood was 0.003 µg/g Pb. The graphs values indicate average ± SD for 5 mice/group; ***p < 0.001 compared with the corresponding control group (ctr), and ^†††^p < 0.001 compared with the corresponding PbO NP group by unpaired t-test. **D** Quantification of total cholesterol, free cholesterol and total CEs analyzed in liver samples measured by Cholesterol/Cholesteryl Ester Quantitation Kit. The values in graphs indicate average ± SD for 4–5 mice/group; ^†^p < 0.05 compared with the corresponding PbO NP group by unpaired t-test. The amount of cholesterols given in µg was normalized to 1 mg of liver (wet weight). **E** LC–MS quantification of selected cholesteryl esters (CEs) analyzed in liver samples of control mice (ctr), mice inhaled PbO NPs (PbO) and clearance group of mice (PbO/cl). The graphs values indicate average ± SD for 4–5 mice/group; *p < 0.05; ***p < 0.001 compared with the corresponding control group (ctr), and ^††^p < 0.01, and ^†††^p < 0.001 compared with the corresponding PbO NP group by unpaired t-test. The absolute abundance of free cholesterol is given in µg/mg wet weight. The value is normalized to signals of deutered internal standard and 1 mg of liver (wet weight). The relative quantitative responses of individual CEs are given as a peak area signal normalized to signal of internal standard and 1 mg of wet weight of liver (expressed as a percentage). **F** Representative extracted ion chromatograms (EIC-MRM) of LC-ESI MS/MS separation of selected lipids and internal deuterated standards used in all experiments. Several abundant species of CEs were extracted from livers of control mice. LC–MS data were obtained by using optimized experimental conditions as described in the "Methods" section. Retention times and typical MRM transitions used for quantification are shown for each compound

For a more detailed analysis of cholesterol level alterations after inhalation of PbO NPs, we performed a quantitative evaluation of the amount of free cholesterol (FC) and cholesteryl esters (CEs) from freshly collected samples of livers (Fig. 7A, B). The mice were again placed in the whole-body inhalation chambers where the concentration of NPs was 1.64 × 10^6^ NPs/cm^3^. The size distribution with respect to the number of particles per unit volume and characterization of PbO NPs is shown in Fig. 7A and Table 3. The groups of animals inhaled clean air (ctr) for a period up to 9 weeks, the other group inhaled air with PbO NPs (PbO) for 9 weeks. Moreover, we added one group inhaling air with PbO NPs for 6 weeks and thereafter clean air for following 3 weeks (PbO/cl), which enabled us to determine possible changes during clearance period (Fig. 7B). The estimated deposited dose over the 9 weeks inhalation period was 0.697 µg of PbO per gram of mouse body weight.

**Table 3:** Characterization of generated PbO NPs (2nd experiment)

Characterization of PbO NPs	PbO
Number concentration	1.64 × 10^6^ NPs/cm^3^
Surface area	6.14 × 10^9^ nm^2^/cm^3^
Mode	31.1 nm
Geometric mean diameter	26.6 nm
Geometric standard deviation	1.70
Mass concentration	75.5 µg PbO/m^3^
Estimated deposited dose (after 9 w)	0.697 µg PbO/g
Estimated deposited dose (after 6 w)	0.465 µg PbO/g

The chemical analysis confirmed high concentration of Pb (0.17 µg/g Pb; p < 0.001 compared with the corresponding control group) in the blood after 9-week PbO NP inhalation (Fig. 7C). Limit of detection for Pb in the blood was 0.003 µg/g Pb. Three-week long clearance period caused a significant decrease of Pb in the blood (0.02 µg/g Pb; p < 0.001 compared with the corresponding PbO NP group).

The amount of both total (TC) and free cholesterol (FC) in the liver tissue was increased after 9-week PbO NP inhalation (albeit not significantly) (Fig. 7D). Although total concentration of CEs was reduced only non-significantly (Fig. 7D), a more detailed LC–MS screen revealed that the levels of selected cholesteryl esters (CE 16:0, 18:1, 20:4, 22:6) in liver significantly decreased after 9-week PbO NP inhalation (*p* < 0.001 compared with the corresponding control group) (Fig. 7E). Three-weeks clearance period following after 6-weeks PbO NP inhalation led to a significant increase of total CEs (*p* < 0.05 compared with the corresponding PbO NP group) (Fig. 7D). This trend was confirmed by LC–MS data, which revealed an increase of all studied cholesteryl esters (CE 16:0, 18:1, 20:4, 22:6) in liver of clearance group compared to control group (*p* < 0.05 or *p* < 0.001) or to corresponding PbO NP group (*p* < 0.01 or *p* < 0.001). As an example, we displayed a result of LC–MS analysis of lipids extracted from liver of control mice, where the chromatographic peaks correspond to FC and individual kinds of CEs species (Fig. 7F).

In summary, the decreased amount of cholesteryl esters as metabolites of cholesterol was observed after PbO NP inhalation in liver together with higher levels of total and free cholesterol. This indicated a disruption of cholesterol homeostasis in the liver in response to PbO NP exposure, which might be linked also with the initiation of liver steatosis [48].

### Effect of PbO NPs on hepatocytes in vitro

**Fig. 8:**
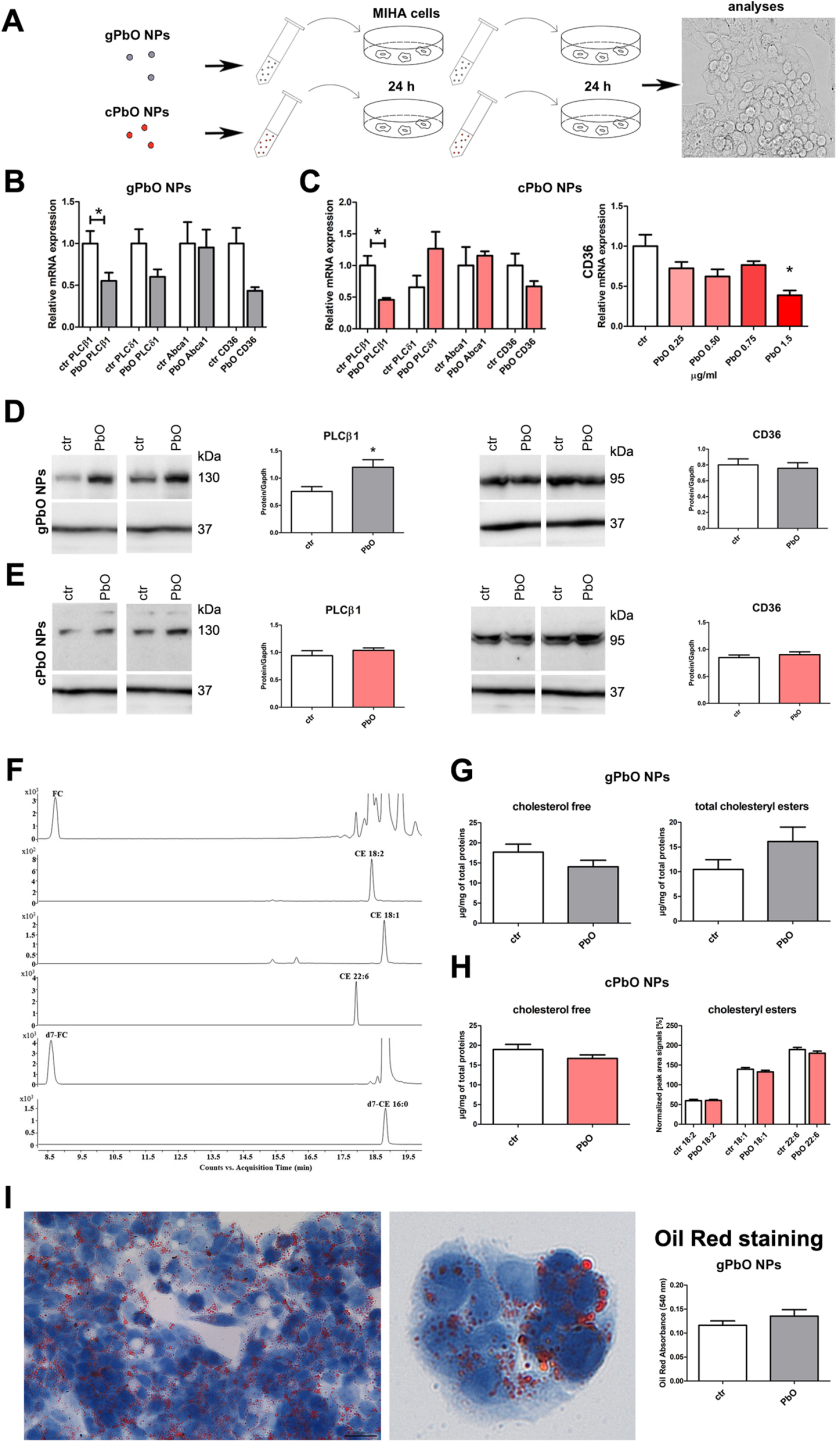
Effect of PbO NPs on liver cells. **A** Design of *in-vitro* experiments. Symbols of gray circle indicate in-situ generated PbO NPs (gPbO NPs), and symbols of red circles indicate commercially available PbO NPs (cPbO NPs). **B** Gene expression of selected phospholipases C, receptors *CD36* and *Abca1* in cells after gPbO NP treatment. The graphs values indicate average ± SD; *p < 0.05 compared with the corresponding control group (ctr) by unpaired t-test. **C** Gene expression of selected phospholipases C, receptors *CD36* and *Abca1* in cells after cPbO NP treatment. The graphs values indicate average ± SD; *p < 0.05 compared with the corresponding control group (ctr) by unpaired t-test. **D** Protein expression of phospholipase PLCβ1 and receptor CD36 after gPbO NP treatment. The quantitative comparison of protein levels was normalized to GAPDH levels. The band densities are representative of three independent experiments. The graphs values denote average ± SD; *p < 0.05 by unpaired t-test. **E** Protein expression of phospholipase PLCβ1 and receptor CD36 after cPbO NP treatment. The quantitative comparison of protein levels was normalized to GAPDH levels. The band densities are representative of three independent experiments. The graphs values denote average ± SD. **F** Representative graphs extracted ion chromatograms (EIC-MRM) of LC-ESI MS/MS separation of selected lipids and internal deuterated standards. Free cholesterol and three most abundant species of CEs were isolated from liver MIHA cells. **G**, **H** Quantification of free cholesterol and total and selected individual CEs analyzed in MIHA cells after gPbO NP or cPbO NP treatment measured by LC–MS and Cholesterol/Cholesteryl Ester Quantitation Kit. LC–MS provided information about changes of free cholesterol and three most abundant species of CEs. The total CEs and free cholesterol concentrations were obtained using the commercial kit. The abundance of free cholesterol is given in µg. The relative quantitative responses of individual CEs are given as normalized peak area signals (expressed as a percentage). All obtained values were normalized to the signals of deuterated internal standards and 1 mg of total proteins in the sample. The statistical significance was evaluated by unpaired *t*-test. **I** Oil Red staining of lipids in MIHA cells and measuring of their absorbance to quantify oil content after gPbO NP treatment

To further investigate the effect of PbO NPs on liver cells, we performed in vitro analyses while using immortalized human hepatocytes—MIHA cell line (Fig. 8). For these experiments, we have used two types of nanoparticles: (1) generated PbO NPs (gPbO NPs; labelled by grey colour) *in-situ* by evaporation of Pb wire and collected into cell culture media; and (2) commercially available PbO NPs (cPbO NPs; labelled by red colour) (Fig. 8A).

The levels of phospholipase *PLCβ1* and receptor *CD36* mRNAs were decreased after gPbO NP as well as cPbO NP treatment (Fig. 8B, C). The mRNA level of transporter *CD36* was significantly lower (*p* < 0.05 compared to control group) after cPbO NP treatment, when using a higher concentration of cPbO NPs (1.5 µg/ml) (Fig. 8C). The mRNA levels of phospholipase *PLCδ1* and transporter *Abca1* were not significantly affected after gPbO NP and cPbO NP exposure (Fig. 8B, C). The basal levels of *PLCγ2* and *Abcg1* mRNAs was so low that we were not able to perform their quantification in any sample.

We then determined also the levels of PLCβ1 and receptor CD36 proteins in MIHA cells exposed to PbO NPs. Irrespectively of down-regulation of their mRNAs, upon the treatment with PbO NPs, the level of phospholipase PLCβ1 protein increased and the level of CD36 remained unchanged (Fig. 8D, E).

Next, we performed the quantitative evaluation of cholesterol level in MIHA cells, in a similar manner as previously mentioned for mice liver tissues. An example of LC–MS chromatogram of lipids extracted from MIHA cells is shown in Fig. 8F. The chromatographic peaks correspond to FC and three most abundant CEs species. The amount of free cholesterol was slightly decreased both after 48 h gPbO NP (17.7 → 14.1 µg/mg total proteins) and cPbO NP treatment (18.8 → 16.7 µg/mg total proteins) (Fig. 8G, H). The total cholesteryl esters were upregulated (10.5 → 16.1 µg/mg total proteins) in this time point/after 48 h gPbO NP treatment; however, these changes were not significantly different when compared with control groups (Fig. 8G).

The amount of three selected cholesteryl esters (CE 18:2, CE 18:1, CE 22:6) after 48 h cPbO NP treatment was slightly lower but it was not statistically significant when compared to control group (Fig. 8H).

Since we observed further morphological changes in liver cells after their exposure to gPbO NPs treatment, we also performed Oil Red staining, in order to evaluate possible accumulation of lipid droplets in their cytoplasm (Fig. 8I). Upon the treatment with gPbO NPs, we observed a minor increase of total amount of lipids in MIHA cells.

### The exposure to PbO NP did not alter spleen macrophage distribution or their morphology

After the 11-week PbO NP inhalation, the concentration of Pb in the spleen was at the same level as in the liver. However, histopathological analysis of the spleens of PbO NP inhaling animals did not reveal any significant change (Fig. S3). The proportion of red and white pulp was about the same in both PbO NP-exposed and control animals. CD68-positive cells were detected predominantly in the red pulp where macrophages typically reside. Collagen fibers were rare in splenic parenchyma in both PbO NP exposed and control animals. The only changes observed in animals exposed to PbO NPs were increased numbers of megakaryoblasts and megakaryocytes in splenic parenchyma (Table S6, Fig. S3, S4), however, these changes were not significant.

## Discussion

Nanoparticles are nowadays routinely applied in almost all segments of human activity, including medicine, pharmaceutical production, food industry, cosmetics, building industry or electrical engineering, and their production is increasing. Nanoparticles are thus becoming an inseparable component of the environment [62], and this includes also lead nanoparticles that are being widely used in the modern industry [9, 26, 31].

A surface area of nanoparticles acquires increasing attention as an important parameter in assessing the toxicity of nanoparticles [63, 78] and recently, it has been presented as the most biologically relevant dose metric for NPs toxicity in the lung [72]. Consequently, the deposition of NPs in the respiratory tract was studied to assess the toxicological impacts of NPs entering the lungs. The aerosol deposition depends on lung morphometry, respiratory physiology, air flow, and particle properties (especially on particle size, shape and density) [50, 81]. To predict particle deposition, various mathematical models have been developed such as ICRP, MPPD etc. [3, 20, 33, 50, 56]. Because of the complexity of deposition processes, all models make simplifying assumptions on lung morphometry and use theoretical or empirical equations for deposition efficiency of airway segments [50, 81]. Here, we used two models, i.e. the ICRP and the MPPD, to predict PbO NP deposition in different regions of respiratory tract. At first, the deposition of PbO NPs in the mouse respiratory tract was calculated using the MPPD model and then the deposition of PbO NPs in the human respiratory tract was calculated using the ICRP model. As it follows from the comparison, the deposition fractions calculated using the MPPD model for the extrathoracic, tracheobronchial, and alveolar regions of the mouse respiratory tract are different from those calculated with the ICRP model for the human respiratory tract. The difference between deposited fractions especially in alveolar and extrathoracic regions of mouse and human respiratory tract are caused by several reasons. The main reason is different morphology of mouse and human respiratory tract, particularly smaller dimensions of mouse upper respiratory tract, resulting in higher deposition of particles in mouse extrathoracic region that was also observed in other studies [3, 30]. Second, the complete model of the respiratory tract of mouse is still not available, which leads to a less accurate predication of local deposition [3, 50, 64, 76, 82]. Third, both models employ different way of local deposition calculation. The depositions in the ICRP model are calculated for all diameters of generated polydisperse NPs using the reference values of fractional deposition in each region of the respiratory tract that are predicted for reference worker with breathing rate of 1.2 m^3^/h (ie normal nose breather). The depositions in the MPPD model are calculated for mouse strain Balb/C using a geometric mean diameter (i.e. 29.7 nm) combined with the geometric standard deviation (i.e. 1.69) that characterizes the size range of generated NPs. These facts together with different degrees of complexity results in different estimates of NPs deposition in the studied regions of mouse and human respiratory tract.

Metal nanoparticles exhibit the ability to easily cross cell membranes in exposed animals [6, 22, 42, 75]. From the lungs, nanoparticles are distributed by blood after a relatively short time to other tissues and organs [7, 24, 25, 37, 69]. Based on data demonstrated previously, we may confirm that nanoparticles pass from the lungs to the blood and are distributed to target organs, where they induce further tissue alterations [23–25].

Most studies evaluating Pb distribution in body organs use administration of Pb in drinking water or food. Upon oral entry, Pb typically accumulates in three compartments: bone, blood, and soft organs. In soft organs, orally administered Pb distributes in the following order kidney → spleen → liver [34, 77]. Deposition of Pb in skeletal elements upon oral as well as inhalation entry is well documented [66], so that here we focused our attention to soft organs. The inhaled Pb nanoparticles were biodistributed and accumulated in soft organs in the same order as upon oral entry: kidney → spleen → liver. The concentration of Pb in the lungs as the primary target organ was practically identical to the kidney. We have seen such similar accumulation of Pb in the lungs and kidney also in our previous study at the end of 11 weeks of inhalation a higher concentration of PbO NPs [41]. In current study, the Pb concentration in the liver and also the spleen was about 3.5-fold lower compared to the lung and kidney. In our previous study [41], where only liver and not spleen was analysed, such drop was about fourfold. The data produced here revealed the increased weight of the lungs and kidneys after subchronic PbO NP inhalation. The increased weight of the lung after PbO NP exposure was also documented by previous studies [8, 65]. Although some small variation in kidney weight could also be due to organ dissection, we are confident that the increase observed here could be caused by NP accumulation in the kidney and the high-volume blood supply of this organ [47]. It is of note that intraperitoneal application of PbO NPs in rats also resulted in increased mass of their kidneys (g kidney per 100 g body mass) [53].

Since Pb is mainly excreted by kidneys [19], the effects of inhalation of PbO NPs to this organ was studied first. Lead toxicity initiates numerous functional changes in the kidney, such as dysfunction of the proximal tubules, and numerous morphological changes to renal tissue, such as development of interstitial fibrosis [44, 66]. In our study, we observed only minor changes in renal tissue after PbO NP inhalation, which is rather unexpected, knowing the highest concentration of Pb found in the kidney (kidney > lung > liver > spleen). Moreover, it should be noted that using TEM, we saw only sporadic PbO nanoparticles in the kidney tissues. Thus, since the high concentration of Pb as measured by chemical analysis did not coincide with low frequency of occurrence of PbO nanoparticles, we assume that most of the Pb in the kidney could be converted to yet unidentified non-particulate form.

Unlike the kidney being free of any obvious inflammatory alterations, the inhalation of PbO NPs caused serious inflammatory changes in the lung tissue similarly as other metal nanoparticles, such as CdO or TiO_2_ [6, 17, 22, 42]. Alveolar macrophages are primary cells that phagocytose pathogenic particles [57]. In this study, the number of macrophages after PbO NP inhalation increased similarly as was previously observed with intratracheal exposure to TiO_2_ or CdO nanoparticles [6, 61]. In contrast, inhalation of soluble Pb nanoparticles represented by Pb(NO_3_)_2_ caused significant decrease in macrophage numbers [24, 25]. Also, other studies using different types of metal NPs (CeO_2_NPs, ZnONPs, NiONPs, and CuONPs) [16, 17] confirmed a major variability in reaction of the lungs towards specific types of NPs.

The presence of foam macrophages in the lung is typical following prolonged exposure to metal NPs, but processes involved in the induction of macrophage transformation into foam cells during inflammatory processes are not so well substantiated [2, 71]. High-fat diet, lipid storage disease, and internalization or degradation of surfactant lipids have all been suggested as potential sources of excessive accumulation of lipids [28]. Because of numerous cholesterol crystals observed in lung macrophages after PbO NP exposure, we have decided to analyse molecules involved in cholesterol metabolism (*CD36*, *SR-A1*, *SR-B1* and *Abca1*), however, the presence of foam macrophages was not accompanied with significant changes of these receptors on mRNA levels only small increase was observed in case of *Abcg1.* As lungs are very heterogenous tissues containing large number of different cell types, it will be necessary to follow these processes in future while using separate pulmonary cell lines and especially focus on alveolar macrophages response to distinct types of nanoparticles. The minor changes in gene expression of scavenger receptors can indicate the existence of additional mechanisms being responsible for development of foam cells in lungs of animals exposed to PbO NPs. Moreover, the balance of functional polarization between M1 (pro-inflammatory and antibacterial) and M2 (anti-inflammatory) macrophages can modify the specific response to nanoparticle stimuli and could affect our results.

Phospholipases C plays important roles in the regulation of both the adaptive and innate immune response of organisms. PLCβ1 is expressed in several different organs including lungs, and it regulates the expression of monocyte chemoattractant protein (MCP-1) [87]. Here, however, increased number of macrophages in the lungs after 11-week inhalation was paralleled by decreased level of PLCβ1 mRNA. This may perhaps reflect reaching of sufficient numbers of macrophages in lungs, in order to maintain homeostasis.

While PLCγ1 is widely expressed in several tissues, PLCγ2 expression is restricted to hemopoietic cells [58]. Members of PLCγ activate both lymphocytes of adaptive immunity and all cell types participating in innate immunity (macrophages, natural killer cells, mast cells, and neutrophils) [4]. The role of PLCγ2 in macrophages is not clear; however, activation of PLCγ2 in mast cells mediates degranulation and cytokine secretion, and in neutrophils, induces adhesion and cell spreading [4]. In our study, we have seen statistically significant increase in expression of PLCγ2 in animals exposed to PbO NPs, which may mediate activation of specific immunity through lymphocytes, as well as activation of additional cell types involved in innate immunity. PLCδ1 is expressed in M1 pro-inflammatory macrophages, but not in M2 [87]. The studies suggest that PLCδ1 negatively regulates the toll-like receptor (TLR) mediated inflammatory response in macrophages. Here, *Plcδ1* mRNA was decreased in animals exposed to PbO NPs so that it may contribute to altered activity of M1 macrophages in their lungs.

After acute PbO NP exposure, the level of PLCβ1 mRNA becomes decreased while the level of PLCβ1 protein becomes up-regulated upon exposure to NPs. Although this may look curious, one may not expect the protein level to always follow the level of its mRNA. There may be a cyclicity in synthesis and degradation of PLCβ1 and/or some influence on PLCβ1 levels due to changes given by in vitro culture rather then NP exposure. Unfortunately, from the available data, we can only speculate about these regulations and this will be necessary to follow in future.

Exposure to metal NPs can trigger toxic effects also in the liver, as documented in previous studies [40, 59], and the effect of PbO NPs on lipid metabolism can be expected [23–25]. On the other hand, histological and ultrastructural changes of the liver as well as biochemical parameters of blood were found to be surprisingly independent of the Pb dose and the length of exposure [35]. In our study, scavenger receptor CD36 (SR-B2) became significantly altered in the liver of animals exposed to PbO NPs, as well as in human immortalized hepatocytes exposed to PbO NPs. Subchronic PbO NP exposure caused increased mRNA expression of *CD36* (cholesterol influx), and reduced expression of *Abca1* (cholesterol efflux), and lipid analysis confirmed higher amount of total and free cholesterols in liver tissue. Interestingly, the similar pattern of mRNA expression of *CD36* (increased) and *Abca1* (reduced) was detected also in the kidney after subchronic PbO NP exposure. On the contrary, acute PbO NP exposure (both gPbO NPs and cPbO NPs) lead to reduced mRNA expression of *CD36* (cholesterol influx), and lipid analysis noticed decreased FC in cells (both after gPbO NP and cPbO NP exposure), and total CEs were increased. Similar effect was observed after exposure to TiO_2_ NPs, when *CD36* mRNA also decreased (CD36 protein level was without significant change) [74]. These findings indicate that metal nanoparticles load on hepatocytes influences the level of cholesterol metabolism on the cellular level. On the other hand, acute exposure to ZnO NPs resulted in intracellular accumulation of lipids combined with increased levels of the receptors SR-A and CD36 (both mRNAs and proteins), which are both involved in the uptake of modified LDL [74]. Taken together, not only the length of metal NP exposure, but also the type of used nanoparticles may determine the impact of nanoparticles on lipid metabolism. Moreover, the tissues are composed from multiple types of cells exhibiting differential alterations in lipid metabolism, which can significantly differ when using just individual cell lines in vitro and this complex tissue response needs to be taken during analyses in to account.

Our results demonstrated specific effect of PbO NPs on cholesterol metabolism in liver tissue as well as in hepatocytes, but these processes have not been confirmed to be involved in lung response to nanoparticle exposure. Thus, the insight into the effect of Pb/PbO NPs on lipid metabolism requires additional research to elucidate events contributing to the initiation of foam macrophages in lungs.

## Conclusion

The most dramatic effect of prolonged subchronic inhalation of PbO nanoparticles was profound alteration in lipid exchange and enhanced cholesterol storage seen in the studied organs. In the lungs, the occurrence of large foam macrophages in the alveolar area and interstitial tissue was observed. Activity of lung macrophages seems to be suppressed as indicated by altered expression of several members of PLC family members, which typically regulate inflammatory response of this cell type. Since these changes are accompanied by a modified expression of scavenger receptors, it is likely that they are due to the disruption of lipid transport across the cell membrane. Disrupted efflux of lipids from the cells and their accumulation in the tissue was also seen in the liver, where it was accompanied by an abnormal expression of cholesterol transporters and phospholipases C. The decreased amount of cholesteryl esters as metabolites of cholesterol together with higher levels of total and free cholesterol in liver may contribute further to alteration of lipid metabolism in the tissues after metal nanoparticles exposure. Overall, this study provides further insight into the structural and regulatory elements underlying an inability of the immune system to properly react to nanoparticle exposure by their removal.

## Material and methods

### Animals

Adult female mice (CD-1(ICR) BR strain) with an average weight of approximately 24 g (about 6–8 weeks old) at the beginning of the inhalation experiment were obtained from the Animal Facility of the Masaryk University (Brno, Czech Republic). Mice were allowed to acclimatise to laboratory conditions one week before the experiment. Commercial feed and drinking water were provided ad libitum. The experiment was approved by the Ethical Board of the Institute of Analytical Chemistry, v.v.i., Czech Academy of Sciences, Brno (Approval No. 64/2016, 15 August 2016).

### Preparation of PbO NPs

PbO NPs were prepared in the same way as in our previous studies [8, 23, 79]. PbO NPs were generated continuously in situ in a hot-wall tube flow reactor using an evaporation-oxidation-condensation technique in which a ceramic crucible containing a small amount of lead wire was placed inside the ceramic work tube of a vertically oriented furnace (Carbolite TZF 15/50/610). The molten lead was evaporated at the centre of the furnace at a temperature of 830 °C. The resulting metal vapour condensed to form Pb NPs that were carried out of the furnace by an inert nitrogen gas stream and diluted with a stream of air, during which the lead was oxidised to lead oxide. Both flow rates were set at 3 L/min using mass flow controllers. The resulting PbO NPs were diluted in the second step by a stream of air (20 L/min) and used for whole-body inhalation experiments.

### Exposure to PbO NPs

Ten experimental adult female mice were continuously exposed to PbO NPs at a concentration of 0.956 × 10^6^ NPs/cm^3^ for 11 weeks (24 h/day, seven days/week) in whole body inhalation chambers as described in previous studies [8, 79, 80]. Ten control animals were exposed to the same air as treated animals but without the addition of PbO nanoparticles. The food for animals was sealed in special boxes protected from the deposition of nanoparticles from the air. Immediately after inhalation exposure (11 weeks long), mice were sacrificed, and the lungs, livers, kidneys, and spleens were weighed and collected for chemical, histopathological, histochemical, ultramicroscopic, immunohistochemical analyses and to study the gene expression of selected markers.

Further, we have performed a new 9-week long experiment with inhalation of PbO NPs. One group of animals inhaled clean air (ctr) for a period up to 9 weeks, the second group inhaled air with PbO NPs (PbO) for 9 weeks, and the third group inhaled air with PbO NPs for 6 weeks and thereafter clean air for following 3 weeks (PbO/cl) (Fig. 7A, B). Immediately after inhalation exposure, mice were sacrificed, and the blood, and livers were weighed and collected for chemical analysis.

### Characterization of generated PbO NPs

The main characteristics of generated PbO NPs are displayed in Tables 1, 3 and Figs. 1A, 7A. The distribution of NPs concerning particle number concentration was continuously measured directly inside the exposure cages using a scanning mobility particle sizer (SMPS; model 3936L72, TSI Inc., Shoreview, MN, USA). The long-term stability of PbO NPs generation was high. The mass concentration of generated PbO NPs was calculated by dividing the mass of PbO NPs collected on the filter by the volume of the air sample that passed through the filter. The mass concentration of PbO NPs was 149.3 µg PbO/m^3^ during the first inhalation experiment, and 75.5 µg PbO/m^3^ during the second experiment.

Generated PbO NPs were sampled on nitrocellulose filters (pore size 1.2 µm, diameter 47 mm, Millipore, Bedford, MA, USA). Filters were dissolved in HNO_3_ using a UniClever microwave mineralizer (Plazmatronika, Wroclaw, Poland), and the Pb content in the sample was determined using an atomic absorption spectrometry (AAS; AAnalyst 600, Perkin Elmer Inc., Shelton, CT, USA).

The size and shape of PbO NPs were characterized by electron microscopy (EM). Immediately after generation at the furnace output, PbO NPs were collected by electrostatic precipitation using a Nanometer aerosol sampler (model 3089, TSI) on EM grids (copper S160-4, 3 mm in diameter, 400 mesh grids, Agar Scientific, Electron Technology, Stansted, Essex, UK). The samples were analyzed using a Magellan 400 L XHR microscope (FEI Company, Hillsboro, OR, USA), operating in the scanning transmission electron microscope (STEM) mode. The STEM results show that the PbO NPs observed in the gas phase by an SMPS were formed from agglomerates (size range approximately 40–50 nm) of primary particles of 0.4–0.5 nm in diameter (Fig. 1).

Chemical composition of PbO NPs and lead content of nanoparticles present in liver was recently verified by X-EDS analysis of NPs that were visible on ultrathin liver sections [23]. PbO NPs were also identified using ICP-MS scanning of liver and kidney [24, 25]. Moreover, ICP-MS distinguished Pb in ionic form from Pb in PbO NPs form. Pb in ionic form resulted from gradual dissolution of PbO NPs directly in liver, kidney or lung or during transport of PbO NPs by blood from lung.

### Sampling of in situ generated PbO NPs into liquid media

The generated PbO NPs (gPbO NPs) dispersed in the air inside the exposure cage were collected using a Condensation Growth Unit-Aerosol Counterflow Two-Jets Unit (CGU-ACTJU) sampler [51] into cell culture media DMEM/F12 and/or GlutaMAX-I medium (31966021; Thermo Fisher Scientific), which were used for the cultivation of liver cells (MIHA cell line). The CGU-ACTJU sampler enabled a continuous quantitative collection of NPs from 10 L of air into 1 mL of liquid medium. The concetrations of gPbO NP ranged in 0.36–0.72 (mean 0.49) μg PbO/ml.

### Usage of commercially available PbO NPs in liquid media

The synthetic PbO NPs (cPbO NPs; Nanochemazone, Edmonton, AB, Canada, NCZ-CP-525/20) were weighed and diluted into cell culture media to prepare several selected concentrations (0.25, 0.50, 0.75, 1.00, and 1.50 μg PbO/ml).

### Cell culture and PbO NP exposure

The immortalized non-tumorigenic human hepatocyte MIHA cell line [11] was obtained from the Genetic Engineering and Gene Therapy Core, Albert Einstein College of Medicine (New York, NY, USA). Cells were grown in DMEM (with GlutaMAX and pyruvate; 31966021, Thermo Fisher Scientific, Waltham, MA, USA) supplemented with 10% FBS (F7524; Sigma-Aldrich), 1% penicillin–streptomycin (P0781-100ML; Merck Life Science). Only the cells at passage number up to 20 were used for the experiments. MIHA cells were plated at the density of 40,000 cells per cm^2^ in 6-well plates, and then allowed to grow for 96 h. At ~ 70–80% confluency, cells were further cultured in cultivation medium containing selected concentrations (0.25, 0.50, 0.75, 1.00 and 1.50 μg/ml) of commercially available PbO NPs or in-situ generated PbO NPs for 24 h. Subsequently, cell medium was replaced by fresh medium with newly prepared PbO NPs to decrease the effect of possible dissolution of PbO NPs during cultivation. After another 24 h, cells were collected for further analyses (total exposure time of 48 h).

### Chemicals for LC–MS analysis

Solvents and chemicals for LC–MS (LC–MS grade), acetonitrile (ACN), 2-propanol (IPA), deionized water, formic acid and ammonium formate were purchased from Honeywell Burdick and Jackson (Seelze, Germany). Chloroform, n-butanol, heptane, and ethyl acetate (HPLC grade) were provided by Supelco, Sigma and Riedel-de-Haen, respectively. Lipid standards, namely cholesterol (= free cholesterol; FC), d7-cholesterol (d7-FC), 16:0 cholesteryl ester (CE 16:0) and 16:0 cholesteryl-d7 ester (d7-CE 16:0) were acquired from Avanti Polar Lipids (Birmingham, AL).

### Harvesting cell samples for LC–MS analysis

The MIHA cells were cultivated as described above. The cells were detached using a trypsin solution. The cell pellets were washed several times with ice-cold phosphate-buffered saline (PBS) to remove extracellular metabolites. After the final washing step, the cells were resuspended in 200 μL of deionized water, and such cell suspensions were immediately frozen and stored at − 80 °C until analysis.

## Sample preparation for LC–MS analysis of lipids

### Extraction of lipids from cell culture

Extraction of lipids from treated or untreated MIHA cells was carried out according to a slightly modified Bu-Me method [45] optimized for our purposes. Specifically, 500 μL of cold n-butanol:methanol (3:1; v/v) mixture containing a mixture of deuterated lipid internal standards was mixed with 200 μL of cell suspension, which was previously lysed by two freeze–thaw cycles (freezing in liquid nitrogen; thawing at 37 °C for 10 min). The mixture was vortexed and then shaken (5 min; 4 °C). Afterwards, 500 μL of heptane:ethyl acetate (3:1) and 500 μL 1% acetic acid were added to the reaction mixture and vortexed again and shaken (5 min; 4 °C). Each sample was centrifuged (10 500 g; 5 min; 4 °C) and the upper phase was collected. The remaining aqueous layer was re-extracted with heptane:ethyl acetate (3:1). The resulting upper organic layers were combined and dried under a N_2_ stream. The lipid extracts were re-dissolved in 200 μL chloroform/methanol 1:1, diluted, and used for LC–MS analysis.

#### Extraction of lipids from mice liver

Liver samples were collected from a control group of mice (ctr) inhaling only clean air, a group of mice inhaling nanoparticles for 9 weeks (PbO NPs) and from a group of animals inhaling nanoparticles for 6 weeks and then clean air for 3 weeks (clearance group, PbO/cl). Liver samples were frozen immediately after collection and stored frozen at − 80 °C until extraction for analysis. For extraction, 50 mg (wet weight) liver sample was homogenized in 500 μL of a cold mixture of n-butanol:methanol (3:1; v/v) containing a mixture of deuterated lipid internal standards with glass beads using a homogenizer MagNa Lyser Instrument (Roche Applied Science, Penzberg, Germany). The tissue homogenate was extracted by the Bu-Me method as described above (in the section “*Extraction of lipids from cell culture*”).

#### Total protein quantification

A standard bicinchoninic acid (BCA) assay was used to determine total protein concentration in cell suspensions. Protein content in unknown samples was determined spectrophotometrically (562 nm; spectrophotometric BioTek’s reader and data analysis software, Gen 5.2.0™) by comparison with bovine serum albumin (BSA) used as a protein standard sample.

#### LC–MS analysis of lipids in MIHA cells and mice liver

An Agilent 1290 Infinity II UHPLC System (Agilent Technologies, Palo Alto, CA, USA) coupled with an electrospray ion source (ESI) and an Agilent 6470 Triple Quadrupole mass spectrometry system was used for LC–MS/MS analysis.

LC separation was performed using a Poroshell 120 EC C18 column (3 × 150 mm); 2.7 μm (Agilent Technologies, USA), heated at 50 °C. A mixture of ACN:H_2_O 60:40 (A) and IPA:ACN 90:10 (B) was used as mobile phases. Both phases contained 0.1% formic acid and 5 mM NH_4_COOH as ionization agents. The flow rate was set at 0.3 mL/min and the injection volume was 5 μL. The elution gradient was carried out by increasing percentage of mobile phase “B” as follows: a linear gradient from 68 to 70% B during 10 min; followed by a steeper linear gradient from 70 to 99% during 5 min; then an isocratic step at 99% for 4 min. The column was then re-equilibrated to initial conditions for 3 min. The total run-time was 22 min.

Specific precursor/product ion pairs (multiple reaction monitoring; MRM) and retention times were used to identify individual cholesterol species.

Quantitative analysis was carried out in a positive MRM ion mode and using two internal standards (d7-FC for FC and d7-CE 16:0 for all cholesteryl esters). The following MS parameters were fixed for all substances: gas temperature: 250 °C; gas flow: 8 L/min; nebulizer: 33 psi; sheet gas temperature: 400 °C, sheet gas flow 12 L/min, capillary voltage: 3000 V, nozzle voltage 300–600 V and fragmentor 116 V.

A summary of precursor and product ions, collision energies and retention times that have been optimized for the individual molecular species of cholesterols and is presented in the Table 4.

**Table 4:** LC–MS analytical parameters for investigated compounds and internal standards used for lipids quantification

Compound name	Retention time (min)	MRM transitions for quantification *Precursor ion → Product ion [m/z]*	Collision energy [V]
FC	8.4	369.3 → 147.1	24
CE 16:0	18.7	642.6 → 369.3	8
CE 18:2	18.3	666.6 → 369.3	10
CE 18:1	18.6	668.6 → 369.3	20
CE 20:4	18.0	690.6 → 369.3	10
CE 22:6	17.8	714.6 → 369.3	30
d7-FC	8.3	376.4 → 147.1	24
d7-CE 16:0	18.7	649.7 → 376.4	8

The concentration of FC in each sample was calculated by interpolation from a calibration curve constructed using standard sample data normalized to the internal standard and consequential normalization to the total protein amount in each cell extract (results are in the units of µg free cholesterol/1 mg of proteins) or wet weight of liver (results in the units of µg free cholesterol/1 mg of wet weight of liver). The characterization of analysed CEs, for which we did not have individual standards, was performed by relative quantification. The relative abundance of individual CEs, given as peak area signals normalized to signal of internal standard (i.e. d7-CE 16:0) and 1 mg wet weight of liver or 1 mg total amount of proteins in cell samples (expressed as a percentage), were used in order to compare the amounts of the individual CE species in the samples.

Agilent MassHunter Acquisition software (version 10.1) and Agilent MassHunter Qualitative Analysis software (version B 07.00) were used for data acquisition and evaluation of the analysis results.

### Analysis of lipids by Cholesterol/Cholesteryl Ester Quantitation Assay Kit

The Cholesterol/Cholesteryl Ester Assay Kit—Detection (ab65359, ABCAM) was used to determine total cholesterol, free cholesterol, and total cholesteryl esters according to the manufacturer's instructions. The color intensity of the reaction product measured at 570 nm (spectrophotometric BioTek’s reader and data analysis software, Gen 5.2.0™) is directly proportional to cholesterol concentration in the sample. This kit was used as an alternative method to compare the results obtained by LC–MS.

### Oil Red staining of MIHA cells

Neutral lipid analyses were performed using Lipid (Oil Red O) Staining Kit (Sigma-Aldrich). Cells were washed twice with PBS, fixed with 4% PFA, and stored at 4 °C. Samples were incubated for 5 min in 60% isopropanol after they were washed twice with distilled water. Isopropanol was then discarded, and 300 μl of Oil Red O Working Solution (3 parts of Oil Red O Stock Solution and 2 parts of distilled water) was added to samples, and plates were incubated for 20 min at room temperature. Oil Red O Working solution was discarded, and cells were washed with distilled water 5 times. Samples used for measuring absorbance of Oil Red O Working Solution: 1000 μl of isopropanol was used for extracting Oil Red O Working solution. Plates were placed on horizontal mixer for 15 min. Absorbance was measured in 3 100 µl technical replicates for a sample in 96-well plate at 540 nm. For microphotographs, samples used for Oil Red O Staining were co-stained with Hematoxylin added to samples for 1 min, and then washed 5 times with distilled water.

### Histological analysis

Samples of target organs (lung, liver, kidney, and spleen) were fixed overnight in 10% buffered neutral formaldehyde at 4 °C. After that, samples were dehydrated using a series of increasing concentrations of ethanol, then immersed in xylene, and embedded in paraffin wax. Serial histological sections of 5 µm thickness were prepared and stained using standard Hematoxylin–Eosin staining. Selected sections were stained by Masson Green Trichrome (lungs, liver, kidney, and spleen) for collagen fiber analysis and/or by Toluidine Blue staining for mastocytes demonstration (lungs). The sections were examined by light microscopy in a blinded fashion by two histologists. We evaluated at least 8–10 slides per organ in five animals from the control group and five animals from the PbO NP treated group and assessed alterations in histopathological changes in whole sections of the lungs, liver, and kidney (Table S1, Table S2, Table S4).

Photos of the samples were taken using a light microscope (Leica DM5000 B, Leica Microsystem GmbH, Vienna, Austria) with a digital colour camera (Leica DFC480, Leica Microsystem GmbH, Vienna, Austria).

### Immunohistochemistry

After deparaffinisation and rehydration of the sections in xylene and decreasing concentrations of ethanol, citrate buffer (pH = 6) was used as a pre-treatment in a 97 °C water bath. After that, sections were incubated with a blocking serum (VECTASTAIN ABC Kit, Rabbit IgG, PK-4001, Vector Laboratories, Burlingame, CA, USA; VECTASTAIN ABC Kit, Mouse IgG, PK-4002, Vector Laboratories, Burlingame, CA, USA) for 20 min at room temperature (RT), and then incubated with the primary antibody (MPO, CD68; detailed information Table S7).

After incubation of biotinylated secondary antibody (VECTASTAIN ABC Kit, Rabbit IgG, PK-4001, Vector Laboratories, Burlingame, CA, USA; VECTASTAIN ABC Kit, Mouse IgG, PK-4002, Vector Laboratories, Burlingame, CA, USA) for 30 min at RT, the peroxidase-conjugated avidin–biotin complex (VECTASTAIN ABC Kit, Rabbit IgG, PK-4001, Vector Laboratories, Burlingame, CA, USA; VECTASTAIN ABC Kit, Mouse IgG, PK-4002, Vector Laboratories, Burlingame, CA, USA) was applied for 30 min at RT. For visualisation of positive cells, chromogen substrate diaminobenzidine (Liquid DAB + Substrate Chromogen System, K3468, DAKO, Carpinteria, CA, USA) was used. Then, we counterstained samples with hematoxylin.

Photos of samples were taken using a light microscope (Leica DM5000 B, Leica Microsystem GmbH, Vienna, Austria) with a digital colour camera (Leica DFC480, Leica Microsystem GmbH, Vienna, Austria). The number of macrophages in the lungs and liver is presented as the mean ± SD. Analyses were performed on four mice per control group and PbO NP group (Table S2, Table S4). The values of CD68+  macrophages were counted per square millimetre. The number of CD68+ macrophages was evaluated from four slides (10 images/slide) in each animal. The total area of analyzed lungs and livers was 3.346 mm^2^ per animal.

### qRT-PCR analysis

For RNA extraction, the RNeasy Plus Mini Kit (Cat. No. 74136, Qiagen, Germantown, MD, USA) was used. Complementary DNA was prepared according to the manufacturer’s instructions using a gb Elite Reverse Transcription Kit (cat. No. 3012, Generi Biotech, CR). qRT-PCR was performed with a LightCycler® 480 (Roche). The number of analyzed cDNA samples was 4–5 for the control or PbO NP group.

Gene expression values were expressed in terms of the threshold cycle normalized to beta-actin (*Actb*; ID Mm00607939_s1) expression. TaqMan® Gene Expression Assays (cat. No. 4351372, Applied Biosystems, USA) for *CD-36* (ID: Mm01135202_g1), *SR-A1* (ID: Mm00491755_m1), *Abca1* (ID: Mm00442646_m1), *Abcg1* (ID: Mm00437390_m1), *SR-B1* (ID: Mm00450234_m1), *PLCβ1* (ID: Mm01329380_m1), *PLCδ1* (ID: Mm01342462_g1), *PLCγ2* (ID: Mm01242530_m1), *Nfκb1* (ID: Mm00476361_m1), *Il1α* (ID: Mm00439620_m1), *Il1β* (ID: Mm01336189_m1), *Tnfα* (Mm_00443258_m1), *Tgfβ1* (ID: Mm03024053_m1) were used, and gene expression was analyzed with the following program: initial activation step at 95 °C for 10 min, followed by 45 cycles at 95 °C for 15 s, and annealing temperature at 60 °C for 60 s.

### Western blot analysis

The MIHA cells were cultivated as described above. Following the treatment, cells were washed twice with PBS, collected using 150 µl of Laemmli Buffer, and then boiled for 10 min at 95 °C.

The dissected livers were washed with ice cold 1X PBS to remove blood and stored at − 80 °C. The tissue samples with additional SDS lysis buffer were homogenized by MagNA Lyser Green Beads (03358941001, Roche, Prague, Czech Republic) using homogenizer. The samples were obtained as supernatant after centrifugation.

Protein concentrations of all samples were determined using DC Protein assay kit (Bio-Rad, USA). Lysates were supplemented with Bromophenol Blue (0.01%) and β-mercaptoethanol (143 mM), and incubated for 5 min at 95 °C. Equal amounts of total protein (10 µg) were separated with SDS-PAGE, and proteins were transferred onto Immobilon-P PVDF Membrane (IPVH00010, Millipore, Germany). The membranes were blocked with 5% non-fat milk in TBST (20 mM Tris–HCl pH 7.2, 140 mM NaCl, 0.1% Tween 20) and incubated with a appopriate primary antibodies diluted in same blocking buffer at 4° C overnight. Then the membranes were incubated with the secondary antibody Anti-Rabbit IgG—Peroxidase antibody produced in goat (A6154, 1:5000, Merck, Prague, Czech Republic) for 1 h at RT and visualized by ECL-Plus reagent (Amersham Pharmacia Biotech, Piscataway, NJ, USA) according to manufacturer’s instructions. Primary antibodies were used as follows: rabbit polyclonal antibody against PlcB1 (ab185724, Abcam, Cambridge, UK), rabbit monoclonal antibody against CD36 (ab252923, Abcam, Cambridge, UK) and rabbit polyclonal antibody against GAPDH (sc-25778, Santa Cruz Biotechnology Heidelberg, Germany). Band intensities were quantified using ImageJ software (National Institute of Health, Bethesda, MD, USA). The amount of the PLCβ1 and CD36 protein was normalized against the GAPDH.

### Transmission electron microscopy (TEM)

Lungs, livers, and kidneys were cut into approximately 1 mm^3^ samples. Tissue samples were fixed in 3% glutaraldehyde for 24 h, rinsed in 0.1 M cacodylate buffer (minimally three times), and post-fixed in 1% OsO_4_ solution for 1.5 h at RT. After washing in cacodylate buffer, all samples were dehydrated in a series of ascending concentrations of ethanol, followed by acetone. The samples were embedded in the epoxy resin Durcupan ACM, followed by polymerisation for three days at 60–80 °C. From selected parts of the samples, ultrathin Sects. (60 nm thick) were prepared for TEM analysis. The sections were cut using an ultramicrotome Leica EM UC6 (Leica Microsystem GmbH, Vienna, Austria) and collected on formvar-coated nickel grids.

Some sections were without following contrast to study nanoparticles in electron microscopy. We analyzed minimally two non-contrasted nickel grids with 3–5 ultrathin sections of lung (or another organ) on each 50 (or 75) mesh square pattern grid, and the other two contrasted nickel grids with 3 sections on each one. The analyzed area demarcated by black lines of electron grid measured at least about 425 µm × 425 µm (resp. 280 µm × 280 µm)—thus, 0.18 mm^2^ (0.08 mm^2^). To observe clusters of nanoparticles, it was possible only when using magnification at least 18 000 × and with the necessity to enlarge images because the ultrathin sections often display many black arteficial features. Thus, we regularly screened tissue sections line by line to detect these nanoparticles. The sections contrasted with uranyl citrate and lead acetate were used also for visualization of the histopathological alterations of tissues. All sections were examined using Morgagni™ 268 TEM (FEI Company, Eindhoven, Netherlands), working at 90 kV and equipped with a Veleta *CCD* camera (Olympus, Münster, Germany) for taking photographs. The designated structures were measured using iTEM software.

### Transmission electron microscopy (TEM)/Energy-dispersive X-ray spectroscopy (EDS)

TEM imaging and data acquisition was done on Jeol JEM-F200 TEM operated at 200 kV. TEM Images were acquired using TVIPS XF 416 CMOS camera, STEM images were acquired using HAADF detector with detecting angle 24.4–89.4 mrad at camera length set to 250 mm. EDS data were acquired using JED 2300 X-ray spectrometer with a single 100 mm^2^ (0.98sr) windowless SDD detector. Presented quantification was calculated from the raw spectra using standardless Zeta factor method embedded in Jeol Analysis Station software.

### Scanning electron microscopy (SEM)/Energy-dispersive X-ray spectroscopy (EDS)

For SEM/EDS analysis in transmission mode, ultrathin sections of liver tissue were directly deposited on the nickel grids without post-section staining. The grids were placed on an aluminium holder and analyzed with SEM TESCAN MAIA3 (Tescan, Brno, Czech Republic) using 30 keV landing energy, equipped with an EDS detector (X-Max Extreme, Oxford Instruments).

### Determination of lead in mouse organs

In the analysis of organs, the weights of individual organs were determined, and the values were recorded for later quantitative evaluation. The individual organs as well as the whole blood samples were decomposed by microwave (MW) assisted digestion in 3 mL of concentrated subboil grade nitric acid (quartz distillation system model MSBQ 2, Maasen, Eningen, Germany). The samples were treated in pre-cleaned quartz tubes of a closed pressurised autoclave system (UltraWave, Milestone s.r.l., Italy). The decomposition program consisted of four steps: 1st step—10 min with temperature ramp between 100 and 120 °C; 2nd step—5 min with temperature ramp between 120 and 200 °C; 3rd step—3 min with temperature ramp between 200 and 250 °C; and 4th step—5 min at 250 °C. After cooling down (a duration of approx. 10 min), digests were quantitatively transferred to high-density polyethene vials, diluted, and adjusted with ultrapure water (Ultra Clear system, SB Barsbüttel, Germany) to the final volume of 10 ml. Simultaneously, blank samples (typically n = 12 per sampling series) were processed analogously.

The content of lead in the digests was determined by electrothermal atomic absorption spectrometry (ET AAS), employing AAnalyst 600 PerkinElmer (USA) instrumentation under recommended conditions. A mixture of ammonium phosphate and magnesium nitrate was used as a combined chemical modifier. The method of standard addition calibration was applied for quantitation.

### Statistical analyses

Statistical analyses were performed with GraphPad Prism 5 (GraphPad Software, Inc., La Jolla, CA, USA). Unpaired Student’s t-tests were used to determine differences between PbO NP and control groups. Results were reported as the mean value ± standard deviation. Values of *p* < 0.05 were considered to be statistically significant.

## Supplementary Information

**Additional file 1.**  Calculation of deposited dose of PbO nanoparticles. **Figure S1**. Analysis of weight of target organs after exposure to PbO NPs. **Table S1**: Pathological changes in kidney 11-week PbO NP inhalation. **Table S2**. Pathological changes in lung after 11-week PbO NP inhalation. **Table S3**: Analysis of macrophage numbers in lungs. **Table S4**: Pathological changes in liver after 11-week PbO NP inhalation. **Figure S2**: Statistical evaluation of histopathological changes in liver after 11 weeks of PbO NP inhalation. **Table S5**: Analysis of macrophage numbers in liver. **Figure S3**: Spleen after 11-week PbO NP inhalation. **Table S6**: Analysis of megakaryoblasts and megakaryocytes in spleen. **Figure S4**: Statistical evaluation of megakaryoblasts and megakaryocytes in spleen. **Table S7**: List of antibodies used for immunohistochemical analysis.

**Additional file 2.** Cell cultures using generated nanoparticles plus analyses of liver tissues (western blot analysis). Cell cultures using commercial nanoparticles plus analyses of liver tissues (western blot analysis).

## Data Availability

This manuscript has not been submitted simultaneously for publication in any other journal, nor have the findings been partially disclosed in any other publication.
