# Supplementary material for: Macrophage-mediated tissue response evoked by subchronic inhalation of lead oxide nanoparticles is associated with the alteration of phospholipases C and cholesterol transporters
Source: Part Fibre Toxicol. 2022 Aug 3;19:52. doi: 10.1186/s12989-022-00494-7 (PMC9351260; doi:10.1186/s12989-022-00494-7)
Supplement: Supplementary file 1 — Additional file 1. Calculation of deposited dose of PbO nanoparticles. Figure S1. Analysis of weight of target organs after exposure to PbO NPs. Table S1: Pathological changes in kidney 11-week PbO NP inhalation. Table S2. Pathological changes in lung after 11-week PbO NP inhalation. Table S3: Analysis of macrophage numbers in lungs. Table S4: Pathological changes in liver after 11-week PbO NP inhalation. Figure S2: Statistical evaluation of histopathological changes in liver after 11 weeks of PbO NP inhalation. Table S5: Analysis of macrophage numbers in liver. Figure S3: Spleen after 11-week PbO NP inhalation. Table S6: Analysis of megakaryoblasts and megakaryocytes in spleen. Figure S4: Statistical evaluation of megakaryoblasts and megakaryocytes in spleen. Table S7: List of antibodies used for immunohistochemical analysis. [file 12989_2022_494_MOESM1_ESM.docx]

Supplementary Information

**Macrophage-mediated tissue response evoked by subchronic inhalation of lead oxide nanoparticles is associated with the alteration of phospholipases C and cholesterol transporters**

Tereza Smutná^1¤†^, Jana Dumková^2¤^, Daniela Kristeková^1,3^, Markéta Laštovičková^4^, Adriena Jedličková^1^, Lucie Vrlíková^1^, Bohumil Dočekal^4†^, Lukáš Alexa^4^, Hana Kotasová^2^, Vendula Pelková^2^, Zbyněk Večeřa^4^, Kamil Křůmal^4^, Jiří Petráš^5^, Pavel Coufalík^4^, Dalibor Všianský^6^, Samuel Záchej^7^, Dominik Pinkas^8^, Jan Vondráček^5^, Aleš Hampl^2^, Pavel Mikuška^4^, Marcela Buchtová^1,3^*

^1^ Laboratory of Molecular Morphogenesis, Institute of Animal Physiology and Genetics, v.v.i., Czech Academy of Sciences, 602 00, Brno, Czech Republic

^2^ Department of Histology and Embryology, Faculty of Medicine, Masaryk University, 625 00, Brno, Czech Republic

^3^ Department of Experimental Biology, Faculty of Science, Masaryk University, 625 00, Brno, Czech Republic

^4^ Department of Environmental Analytical Chemistry, Institute of Analytical Chemistry, v.v.i., Czech Academy of Sciences, 602 00, Brno, Czech Republic

^5^ Department of Cytokinetics, Institute of Biophysics, v.v.i., Czech Academy of Sciences, 612 65, Brno, Czech Republic

^6^ Department of Geological Sciences, Faculty of Science, Masaryk University, 625 00, Brno, Czech Republic

^7^ TESCAN Brno, s. r. o., 623 00, Brno, Czech Republic

^8^ Electron Microscopy Core Facility of the Microscopy Centre, Institute of Molecular Genetics, v.v.i., Czech Academy of Sciences, 142 20, Prague, Czech Republic

¤ These authors contributed equally to this work

^†^ Deceased

* Correspondence: Marcela Buchtová, Institute of Animal Physiology and Genetics of the Czech Academy of Sciences, Veveří 97, Brno, Czech Republic

Email: [buchtova@iach.cz](mailto:buchtova@iach.cz)

**Calculation of deposited dose of PbO nanoparticles**

The estimation of deposited dose was calculated based on previously published methodology (1, 2, 3) and based on the average mass concentration of PbO nanoparticles (149.3 μg PbO/m^3^).

Deposited dose = (C *RMV * T * DF)/BW (3),

where C is average concentration in the exposure atmosphere 149.3 µg PbO/m^3^ (149.3 ng PbO/L). RMV is respiratory minute volume (L/min) that can be calculated using the equation

RMV =0.499 * BW^0.809^ L/min (1). BW is average body weight (0.024 kg).

T is exposure time (min) equal to 110 880 min (11 × 7 × 24 × 60) for inhalation group (11 weeks). DF is pulmonary deposition fraction (10%), therefore 0.1 (2).

Estimated deposition dose of PbO was 1.684 µg per gram of mouse body weight over the 11 weeks inhalation period.

**References:**

(1) Bide, R. W., Armour, S. J., & Yee, E. (2000). Allometric respiration/body mass data for animals to be used for estimates of inhalation toxicity to young adult humans. *J Appl Toxicol, 20*(4), 273-290.

(2) Miller, F. J. (2000). Dosimetry of particles in laboratory animals and humans in relationship to issues surrounding lung overload and human health risk assessment: a critical review. *Inhal Toxicol, 12*(1-2), 19-57.

(3) Mitchell, L. A., Gao, J., Wal, R. V., Gigliotti, A., Burchiel, S. W., & McDonald, J. D. (2007). Pulmonary and systemic immune response to inhaled multiwalled carbon nanotubes. *Toxicol Sci, 100*(1), 203-214.

**
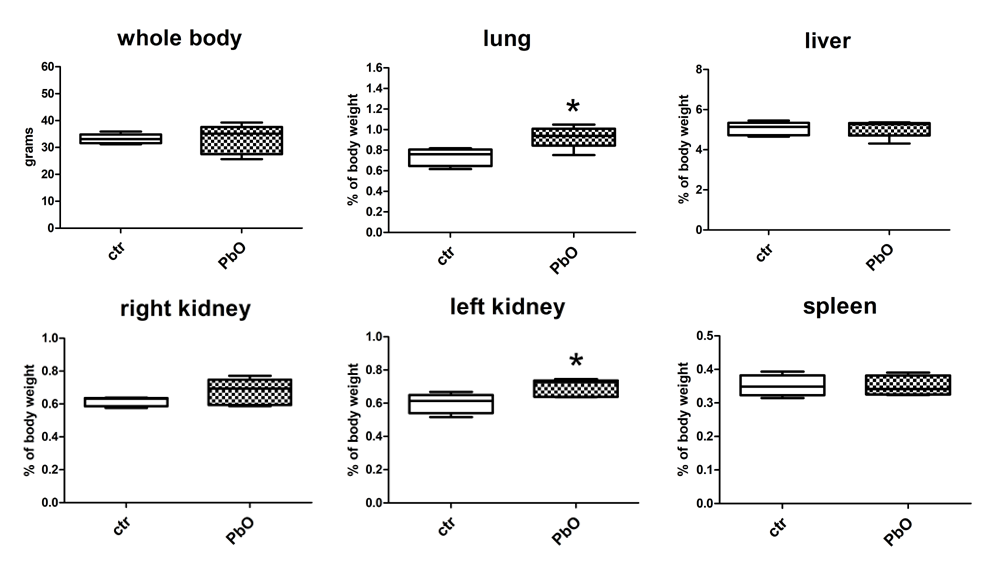
**

**Figure S1. Analysis of weight of target organs after exposure to PbO NPs**. The graphs values denote average ± SD; * p < 0.05 by unpaired t-test.

**Table S1. Pathological changes in kidney after 11-week PbO NP inhalation**

| kidney | control/11w | | | | | PbO/11w | | | | |
| --- | --- | --- | --- | --- | --- | --- | --- | --- | --- | --- |
|  | ctr1 | ctr2 | ctr3 | ctr4 | ctr5 | Pb1 | Pb2 | Pb3 | Pb4 | Pb5 |
| infl. cell inf. perivascular |  |  |  |  |  |  |  |  | + |  |
| infl. cell inf. peritubular |  |  |  |  |  |  |  |  |  |  |
| subcapsular infiltrate |  |  |  |  |  |  |  |  |  |  |
| enlarged JM glomeruli |  | + |  |  |  |  |  |  |  |  |
| glomerular metaplasia | + | + |  |  |  |  |  | + | + | + |
| higher glom. cellularity |  |  |  |  |  |  |  |  |  |  |
| dilation of prox. tubules |  |  |  |  |  |  | ++ |  |  |  |

We evaluated at least 8-10 slides per organ and assessed alterations in histopathological changes as follows - inflammatory cell infiltrate perivascular, inflammatory cell infiltrate peritubular, subcapsular infiltrate, enlarged juxtaglomerular glomeruli, glomerular metaplasia, higher glomerular cellularity, and dilation of proximal tubules. Increased level of phenotype was labelled by increased number of + symbols, where "+" means mild phenotype and "++" moderate phenotype in relevant type of alteration in organ; ctr1 – ctr5 control animals, Pb1 – Pb5 exposed animals.

**Table S2. Pathological changes in lung after 11-week PbO NP inhalation**

| lung | control/11w | | | | | PbO/11w | | | | |
| --- | --- | --- | --- | --- | --- | --- | --- | --- | --- | --- |
|  | ctr1 | ctr2 | ctr3 | ctr4 | ctr5 | Pb1 | Pb2 | Pb3 | Pb4 | Pb5 |
| infl. cell inf. peribron. |  | + | + | + | + | ++ | + | + | + | ++ |
| infl. cell inf. perivasc. |  |  |  |  |  | + | + | + | + | + |
| atelectasis |  |  |  |  |  |  | + |  | + |  |
| bronchiolitis | + |  |  |  |  | + | + |  | + | + |
| thickened septa | + | ++ | + | + | + | ++ | ++ | + | + | + |
| alveolar emphysema |  |  | + |  |  | + |  |  | + | + |
| hemostase |  |  |  |  |  | + |  | + | + |  |
| foam macrophages |  |  |  |  |  | + |  | + | + | + |
| hemorrhage |  |  |  |  | + | + | + | ++ |  | + |

We evaluated at least 8-10 slides per organ and assessed alterations in histopathological changes as follows – inflammatory cell infiltrate peribronchiolar, inflammatory cell infiltrate perivascular, atelectasis, bronchiolitis, thickened alveolar septa, alveolar emphysema, hemostase, foamy macrophages. Increased level of phenotype was labelled by increased number of + symbols, where "+" means mild phenotype and "++" moderate phenotype in relevant type of alteration in organ; ctr1 – ctr5 control animals, Pb1 – Pb5 exposed animals.

**Table S3. Analysis of macrophage numbers in lungs**

|  |  | number of macrophages/ slide | number of foam macrophages/ slide | number of macrophages/ mm^2^ | number of foam macrophages/ mm^2^ |
| --- | --- | --- | --- | --- | --- |
| ctr/11w /  1 | range  mean  SD | 20.5-23.2  **21.88**  7.44 | 1.0-1.3  **1.15**  1.2 | 261 | 14 |
| ctr/11w /  2 | range  mean  SD | 19.2-23.2  **21.28**  6.92 | 0.6-0.8  **0.68**  0.9 | 254 | 8 |
| ctr/11w /  3 | range  mean  SD | 16.0-19.4  **17.68**  5.43 | 0.4-1.1  **0.75**  0.9 | 211 | 9 |
| ctr/11w /  4 | range  mean  SD | 15.6-18.6  **17.00**  5.49 | 0.5-0.7  **0.6**  0.9 | 203 | 7 |
| PbO/11w/  1 | range  mean  SD | 28.7-34.6  **31.95**  8.46 | 4.4-8.2  **6.4**  4.4 | 382 | 76 |
| PbO/11w/  2 | range  mean  SD | 22.0-24.0  **23.40**  6.75 | 5.4-8.5  **6.7**  3.3 | 280 | 80 |
| PbO/11w/  3 | range  mean  SD | 20.7-24.0  **22.28**  5.97 | 3.1-4.7  **3.8**  4.3 | 266 | 45 |
| PbO/11w/  4 | range  mean  SD | 29.7-34.4  **31.55**  6.89 | 6.2-7.4  **6.7**  3.7 | 377 | 79 |

Data are presented as mean ± SD; analyses were performed with four mice per each group. Number of CD68+ macrophages, and foam macrophages was evaluated from four slides (10 images/1 slide) of each animal. The values of CD68+ macrophages were counted per square millimeter.

**Table S4. Pathological changes in liver after 11-week PbO NP inhalation**

| liver | control/11w | | | | | PbO/11w | | | | |
| --- | --- | --- | --- | --- | --- | --- | --- | --- | --- | --- |
|  | ctr1 | ctr2 | ctr3 | ctr4 | ctr5 | Pb1 | Pb2 | Pb3 | Pb4 | Pb5 |
| mononuclear cell inf. |  | + | + | + | + |  | + | + | + | + |
| focal necrosis |  |  |  |  |  |  | ++ | + | + |  |
| polynuclear hepatocytes |  |  |  |  |  |  |  |  |  |  |
| steatosis macrovesicular |  |  |  |  |  |  |  |  |  |  |
| hemostase | + | + | + |  |  | + | + |  | ++ | + |
| hepatic remodeling |  |  | + |  |  |  | + |  | + |  |
| hypertrophic hep. | + |  | ++ |  |  |  |  |  |  |  |
| infiltrate in portal area | + |  |  |  |  |  | + |  | + | + |

We evaluated at least 8-10 slides per organ and assessed alterations in histopathological changes as follows – mononuclear cell infiltrate, focal necrosis (degenerating hepatocytes), polynuclear hepatocytes, macrovesicular steatosis, hemostase, hepatic remodeling, hypertrophic hepatocytes and infiltrate in portal area. Increased level of phenotype was labelled by increased number of + symbols, where "+" means mild phenotype and "++" moderate phenotype in relevant type of alteration in organ; ctr1 – ctr5 control animals, Pb1 – Pb5 exposed animals.


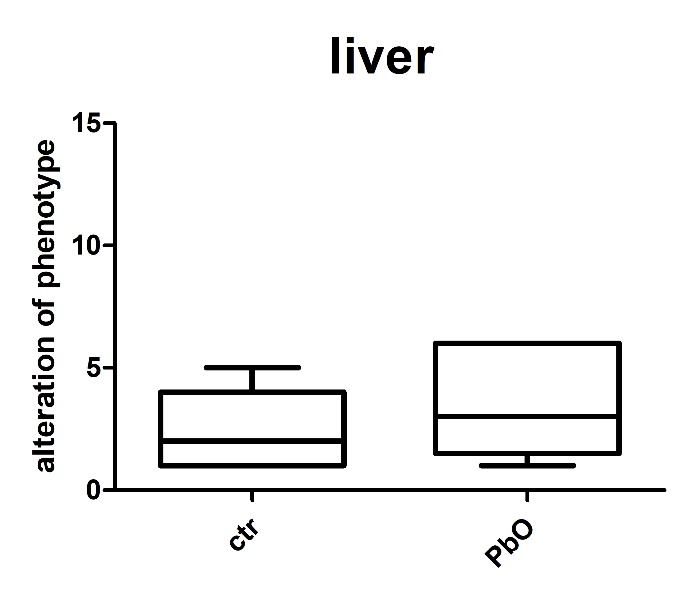


**Figure S2. Statistical evaluation of histopathological changes in liver after 11 weeks of PbO NP inhalation.** Data were obtained according to the **Table S4**. The changes were not statistically significant. The graphs values denote average ± SD.

**Table S5. Analysis of macrophage numbers in liver**

|  |  | number of macrophages/ slide | number of macrophages/ mm^2^ | mean number of macrophages/ mm^2^ |
| --- | --- | --- | --- | --- |
| ctr/11w /1 | range  mean  SD | 35.8-44.6  **41.58**  8.29 | 497 | **511.79** |
| ctr/11w /2 | range  mean  SD | 36.2-42.2  **38.48**  6.63 | 460 |  |
| ctr/11w /3 | range  mean  SD | 37.1-53.1  **45.20**  11.21 | 540 |  |
| ctr/11w /4 | range  mean  SD | 40.0-49.4  **46.00**  7.79 | 550 |  |
| PbO/11w/1 | range  mean  SD | 39.8-53.5  **48.60**  10.31 | 581 | **578.73*** |
| PbO/11w/2 | range  mean  SD | 43.5-53.4  **49.00**  8.86 | 586 |  |
| PbO/11w/3 | range  mean  SD | 45.1-51.6  **48.80**  10.61 | 583 |  |
| PbO/11w/4 | range  mean  SD | 42.4-50.8  **47.25**  8.99 | 565 |  |

Data are presented as mean ± SD; analyses were performed with four mice per each group. Number of CD68+ cells was evaluated from four slides (10 images/1 slide) of each animal, the values of cells were counted per square millimeter; *p < 0.05 compared with the control group (ctr) by unpaired t-test.


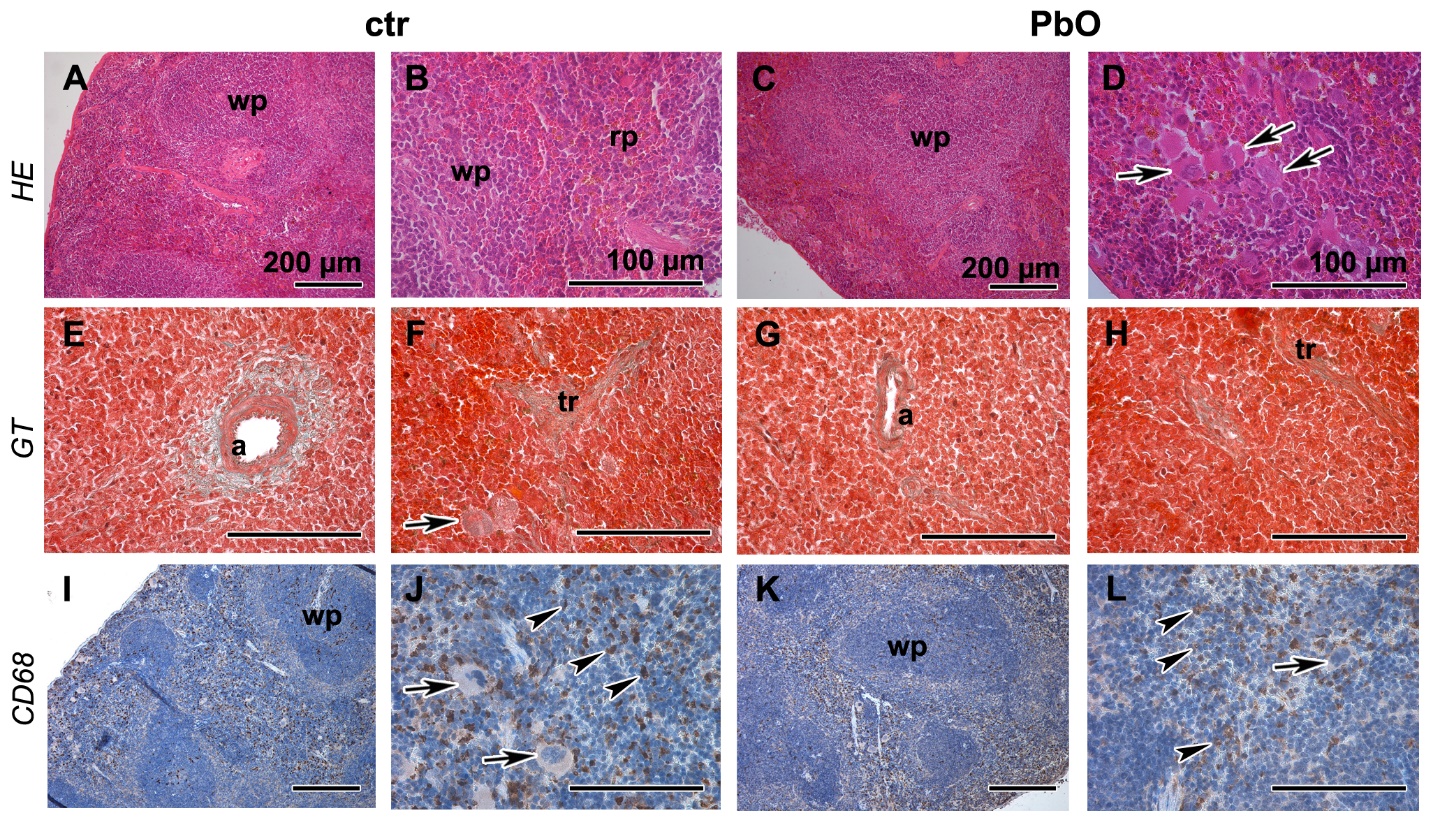


**Figure S3. Spleen after 11-week PbO NP inhalation**

**A, B)** Spleen of control animals (HE staining). **C, D)** Spleen after exposure to PbO NPs; **rp** – red pulp, **wp** – white pulp (HE staining). No significant changes were observed. Scale bars (100 µm or 200 µm) are displayed individually for each picture. **E-H)** Collagen fibers (green) around central arteries (**a**) or inside splenic trabecules (**tr**) in control and PbO NP treated animals (Green Trichrome staining, GT). Scale bars in these panels 100 µm**. I, J)** CD68-positive cells labelled as typical macrophages in controls (*arrowheads*). **K, L**) CD68-positive cells after exposure to PbO NPs (*arrowheads*). Scale bars (100 µm or 200 µm) are displayed individually for each picture similarly as in the panels A-D.

Arrows (**D, F, J, L**) show megakaryoblasts or megakaryocytes in the panels.

**Table S6. Analysis of megakaryoblasts and megakaryocytes in spleen**

|  |  | number of cells/ slide | number of cells/ mm^2^ | mean number of cells/ mm^2^ |
| --- | --- | --- | --- | --- |
| ctr/11w /1 | range  mean  SD | 3.1-3.9  **3.5**  2.8 | 41.8 | **48.3** |
| ctr/11w /2 | range  mean  SD | 4.9-6.2  **5.6**  4.1 | 66.3 |  |
| ctr/11w /3 | range  mean  SD | 2.9-6.7  **4.8**  4.6 | 57.4 |  |
| ctr/11w /4 | range  mean  SD | 1.9-2.7  **2.3**  2.4 | 27.5 |  |
| PbO/11w/1 | range  mean  SD | 2.1-2.5  **2.3**  1.9 | 27.5 | **62.9** |
| PbO/11w/2 | range  mean  SD | 3.8-5.5  **4.7**  3.0 | 55.6 |  |
| PbO/11w/3 | range  mean  SD | 9.8-10.7  **10.3**  5.6 | 122.5 |  |
| PbO/11w/4 | range  mean  SD | 3.2-4.5  **3.9**  2.3 | 46.0 |  |

Data are presented as mean ± SD; analyses were performed with four mice per each group. Number of megakaryoblasts and megakaryocytes was evaluated from two slides (10 images/1 slide) of each animal, the values of cells were counted per square millimeter.


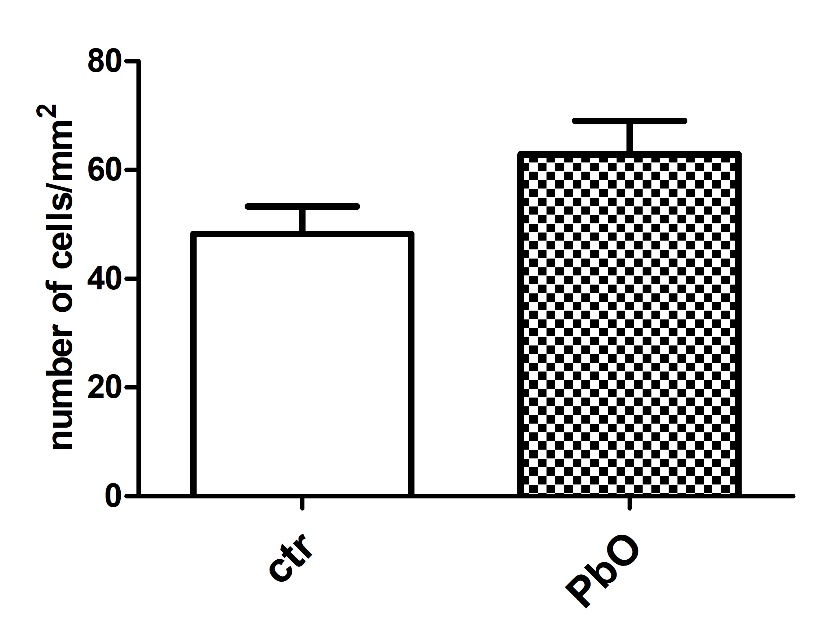


**Figure S4. Statistical evaluation of megakaryoblasts and megakaryocytes in spleen.** The graphs values denote average ± SD after 11 weeks of PbO NP inhalation according to the **Table S6**.; the changes were not statistically significant by unpaired t-test.

**Table S7.** List of antibodies used for immunohistochemical analysis

| **Primary antibody** | **Company** | **Catalog no.** | **Host species** | **Organs** | **Dilution** | **Time/temperature** |
| --- | --- | --- | --- | --- | --- | --- |
| MPO | Abcam | ab9535 | rabbit | lung | 1:50 | 60 min/RT |
| CD68 | Abcam | ab125212 | rabbit | lung, liver | 1:100 | 60 min/RT |
