# Supplementary material for: Macrophage-mediated tissue response evoked by subchronic inhalation of lead oxide nanoparticles is associated with the alteration of phospholipases C and cholesterol transporters
Source: Part Fibre Toxicol. 2022 Aug 3;19:52. doi: 10.1186/s12989-022-00494-7 (PMC9351260; doi:10.1186/s12989-022-00494-7)

Cell cultures using generated nanoparticles plus analyses of liver tissues

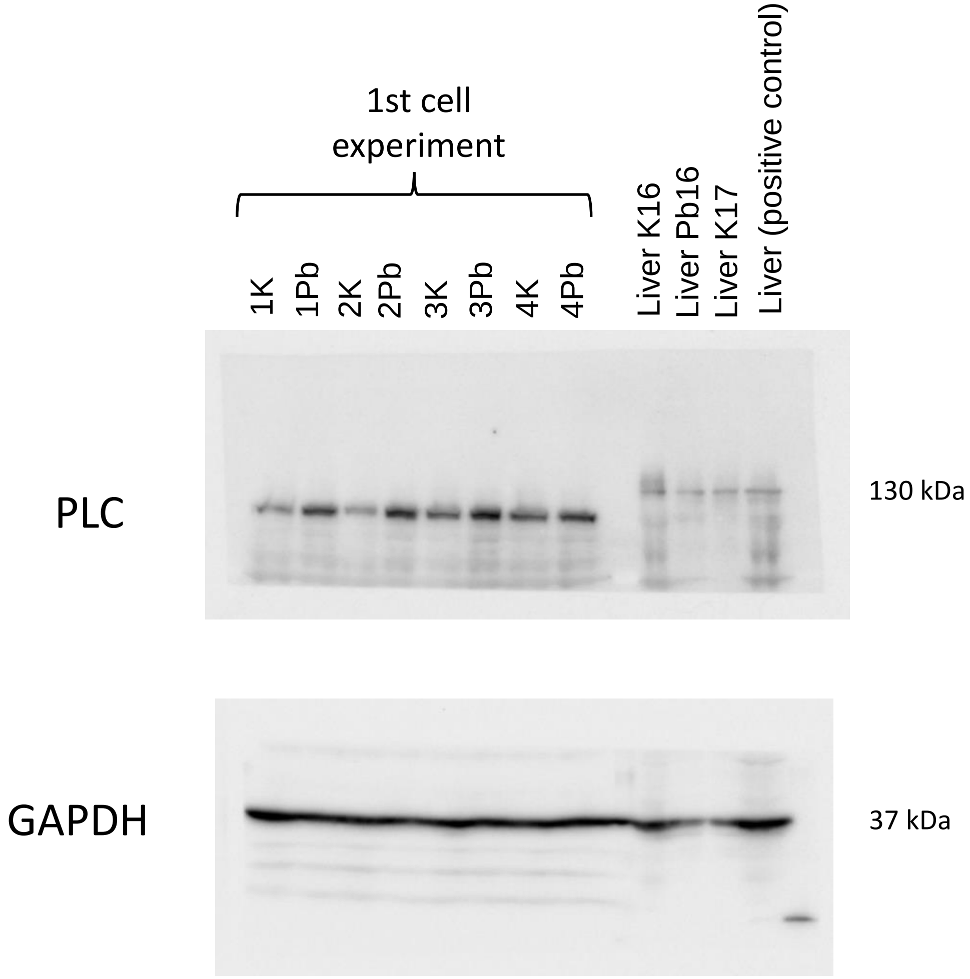

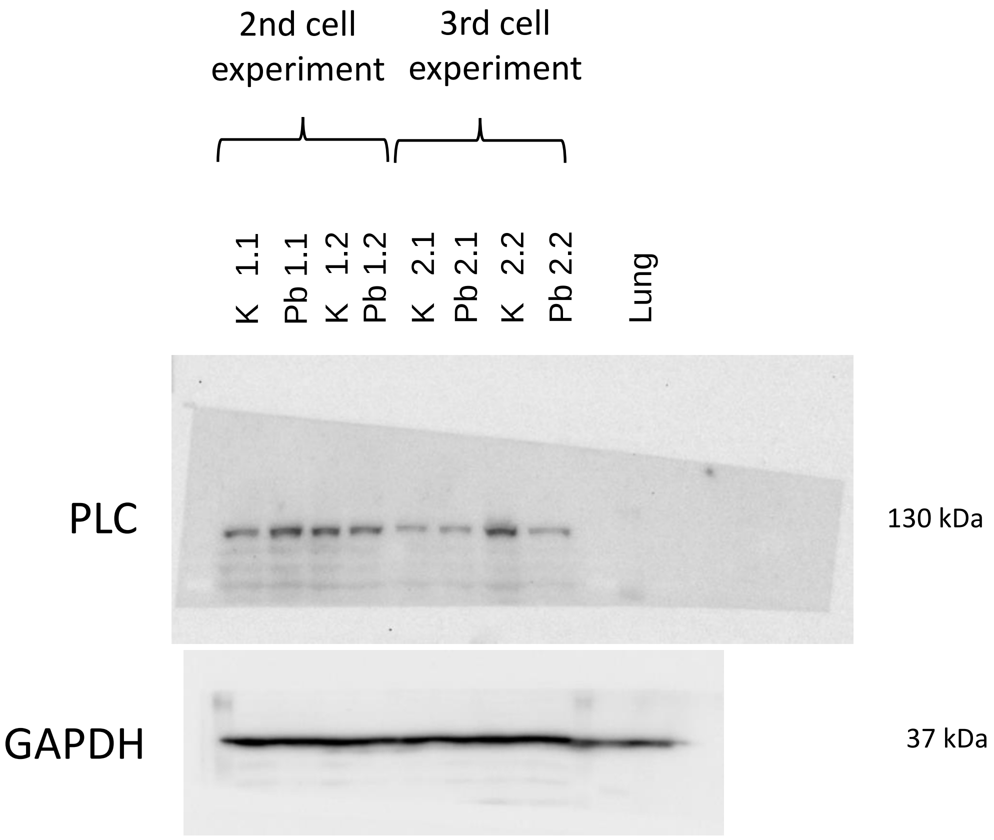

Cell cultures using generated nanoparticles plus analyses of liver tissues

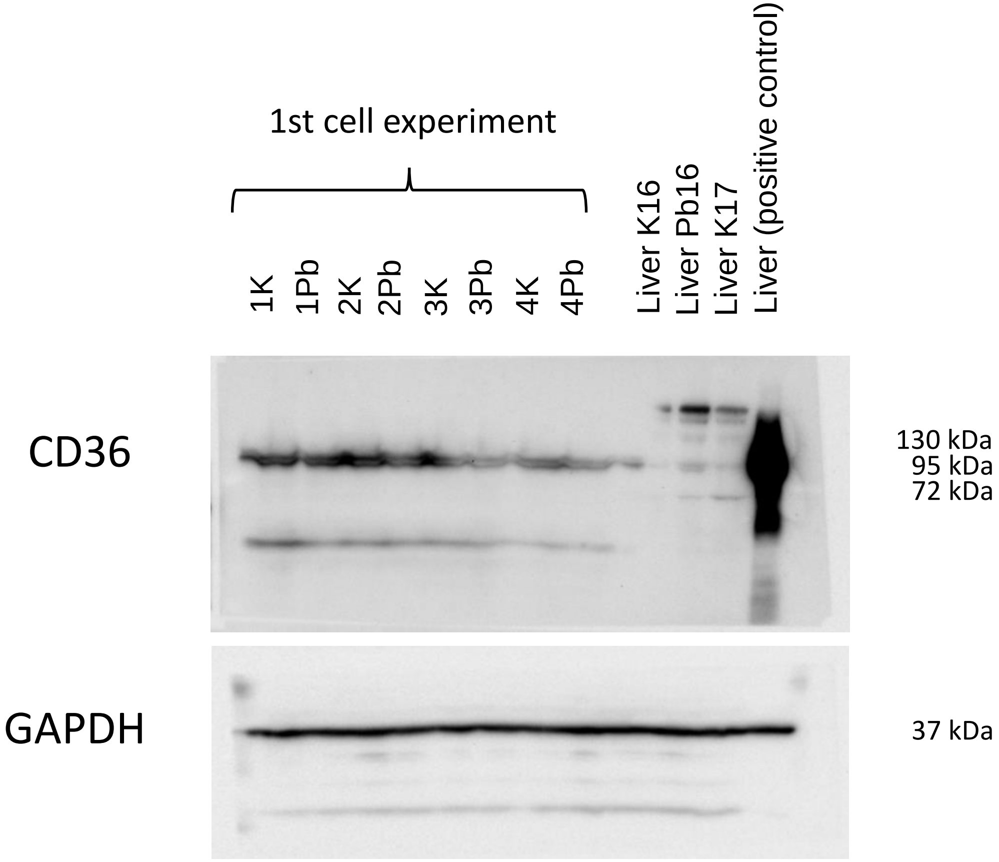

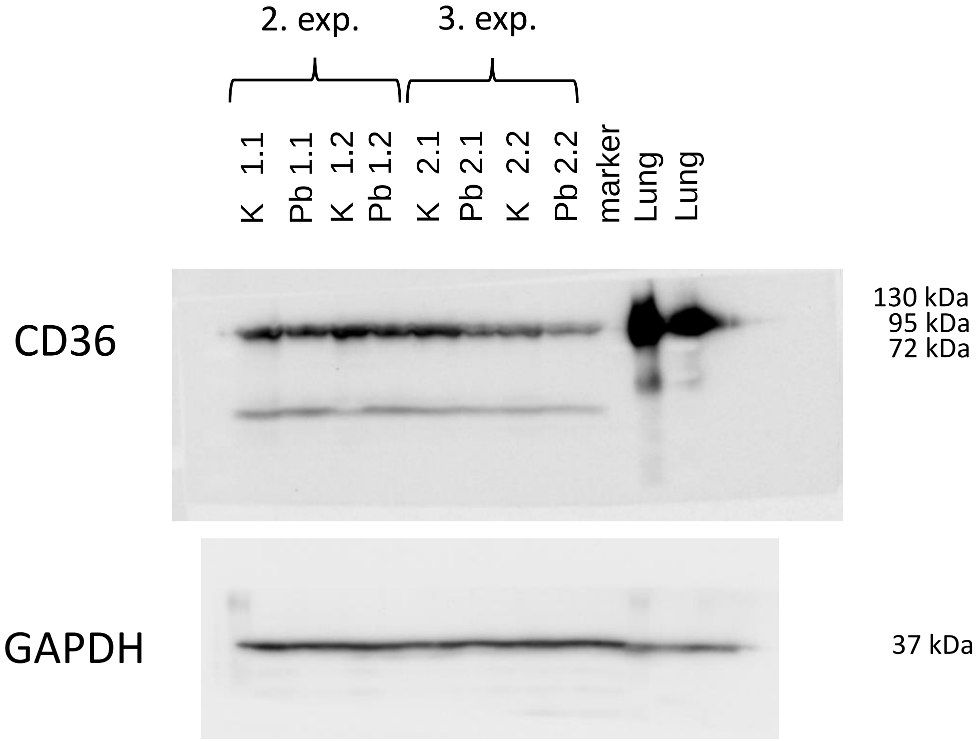

Cell cultures using commercial nanoparticles plus analyses of liver tissues

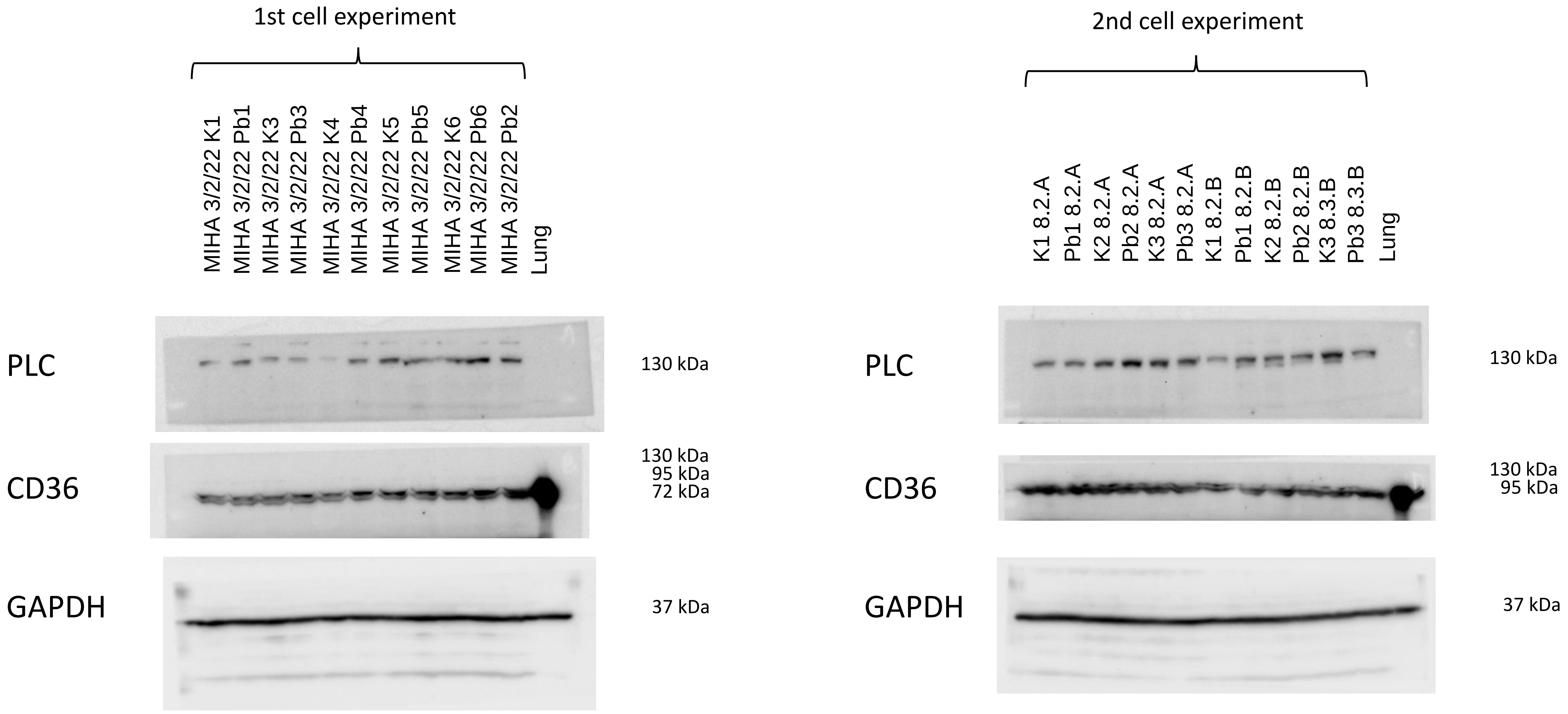

Cell cultures using commercial nanoparticles plus analyses of liver tissues

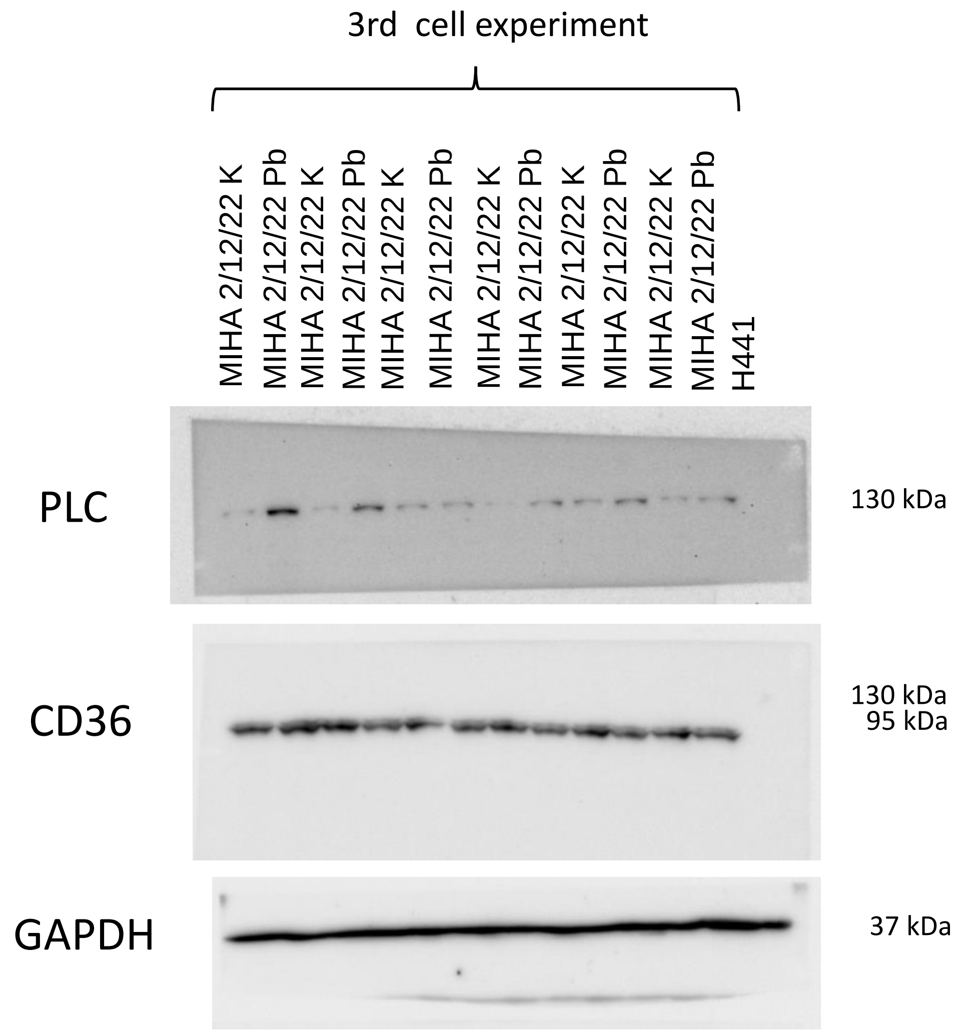

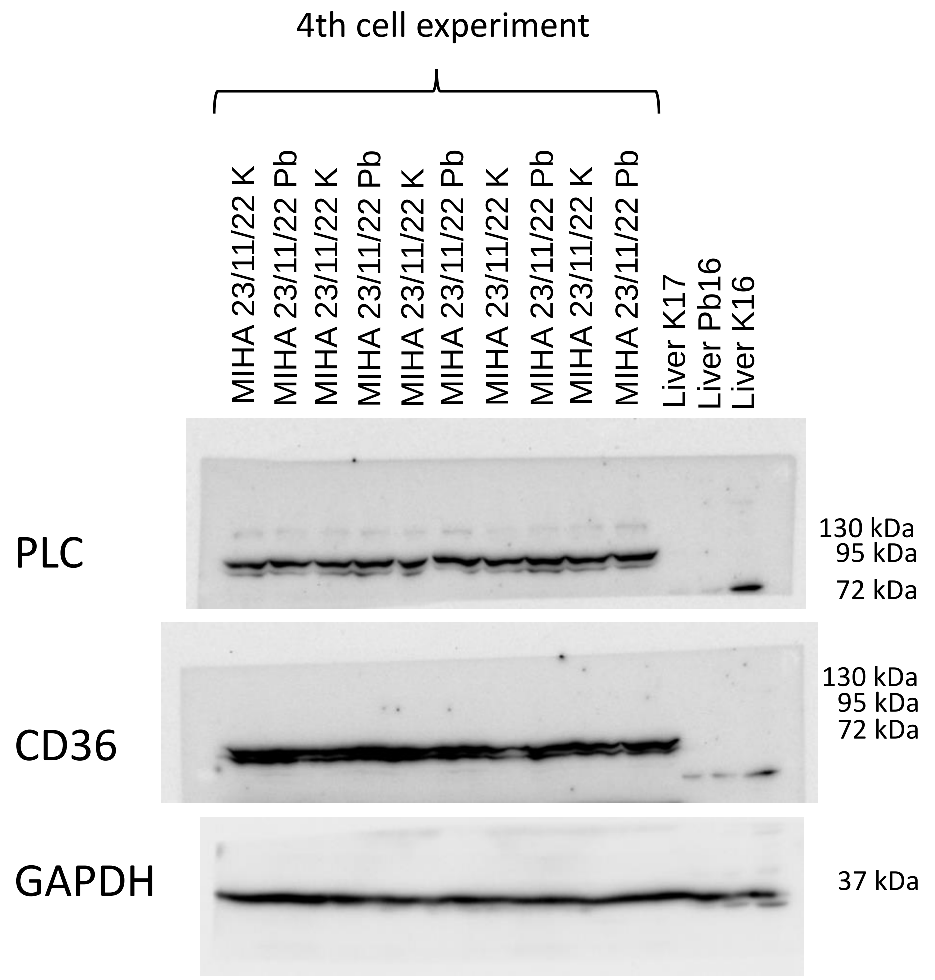

Supplement: Supplementary file 2 — Additional file 2. Cell cultures using generated nanoparticles plus analyses of liver tissues (western blot analysis). Cell cultures using commercial nanoparticles plus analyses of liver tissues (western blot analysis). [file 12989_2022_494_MOESM2_ESM.pdf]
